# Dairy Propionibacteria: Probiotic Properties and Their Molecular Bases

**DOI:** 10.3390/biom15060886

**Published:** 2025-06-17

**Authors:** Franca Rossi, Serena Santonicola, Valerio Giaccone, Alessandro Truant, Giampaolo Colavita

**Affiliations:** 1 Dipartimento di Medicina e Scienze della Salute “V. Tiberio”, Università degli Studi del Molise, 86100 Campobasso, Italy; serena.santonicola@unimol.it (S.S.); colavita@unimol.it (G.C.); 2 Dipartimento di Medicina animale, Produzioni e Salute, Università di Padova, Agripolis, Viale dell’Università 16, 35020 Legnaro, Italy; valerio.giaccone@unipd.it (V.G.); alessandro.truant@unipd.it (A.T.)

**Keywords:** dairy propionibacteria, *Propionibacterium freudenreichii*, *Acidipropionibacterium* species, disease mitigation, immunomodulation, short-chain fatty acids, S-layer proteins, extracellular vesicles, beneficial metabolites, safety

## Abstract

This review summarizes the current knowledge on the probiotic characteristics of dairy propionibacteria, represented by *Propionibacterium freudenreichii* and some *Acidipropionibacterium* species commonly consumed through raw milk cheese. For example, in Swiss-type cheeses, *P. freudenreichii* is added as a starter culture. Some strains of *P. freudenreichii* have been included in mixed probiotic commercial preparations or used to produce tablets from fermented culture media containing bioactive substances such as short-chain fatty acids (SCFAs), bifidogenic molecules, and vitamins. *Acidipropionibacterium acidipropionici* and *A. jensenii* strains have mainly been evaluated as health and productivity promoters in farm animals. For *P. freudenreichii*, the molecular mechanisms behind its probiotic action have been well elucidated, and recently, novel potential applications have been demonstrated in animal models. *P. freudenreichii* strains have been shown to mitigate inflammatory bowel diseases (IBDs) and mucositis and prevent necrotizing enterocolitis (NEC) in newborns. Their immunomodulation capacity has alleviated symptoms of food allergies, obesity, diabetes, colorectal cancer (CRC), and infections. Moreover, *P. freudenreichii* inhibited osteoclastogenesis in a rheumatoid arthritis model. Most observed effects are mediated by proteins on the cell surface or contained in extracellular vesicles (EVs) such as the surface layer (S-layer) protein SlpB, DlaT, and GroEL. No safety issues have been reported for these bacteria. However, investigations into transferable antibiotic resistance traits are still needed, and clinical trials are required to evaluate their effectiveness as probiotics for humans.

## 1. Introduction

Dairy propionibacteria are a taxonomically heterogeneous group of actinomycetes, i.e., high G+C Gram-positive bacteria, which are associated with food and feed environments belonging to the genera *Propionibacterium* and *Acidipropionibacterium*. These two genera were previously classified as *Propionibacterium*, a genus that once comprised food-associated species and cutaneous species of clinical significance. The currently recognized species of propionibacteria associated with food products are *Propionibacterium freudenreichii*, *Acidipropionibacterium acidipropionici*, *A. jensenii*, and *A. thoenii*, and the species less frequently isolated are *Propionibacterium cyclohexanicum*, *A. microaerophilum*, *A. damnosum*, *A. olivae*, *A. virtanenii*, and *A. timonense*. The subspecies of *P. freudenreichii*, *P. freudenreichii* subsp. *freudenreichii* and subsp. *shermanii*, previously separated based on lactose fermentation and nitrate-reducing ability, are no longer considered valid. Indeed, whole-genome sequencing (WGS) highlighted that the first trait is encoded by an integrative–conjugative element (ICE) acquired by horizontal gene transfer (HGT), while the second trait is inactive in some strains due to a frameshift mutation [1,2,3,4].

Dairy propionibacteria are rod-shaped, either anaerobic or microaerophilic, and aerotolerant. Aerotolerance is conferred by a superoxide dismutase, a catalase, the cytochrome *bd* oxidase, heme biosynthesis proteins, and functional electron transport chains. Some strains can grow in aerobic conditions. Their central metabolism produces propionate, acetate, and CO_2_ from lactate via the Wood–Werkman cycle, which involves succinate decarboxylation. These bacteria utilize various carbohydrates, including mannose, arabinose, xylose, and glycerol [5,6,7]. *P. freudenreichii* typically produces mixtures of short-chain fatty acids (SCFAs) in which the amount of propionate is typically at least twice that of acetate [8,9].

Dairy propionibacteria have been isolated from milk, acid whey, traditional cheeses, feed flour, silage with or without grains, soil, fermented vegetables, barley grains, goat rumen, chicken intestine, and fecal samples from breast-fed preterm infants [9,10,11,12,13,14,15]. One study indicated that they survive transit in the gastrointestinal tract when supplied via cheese [16]. Dairy propionibacteria, mainly *P. freudenreichii*, play an essential role in Swiss-type cheese characteristics, forming flavors and distinctive “eyes” via CO_2_ accumulation. In other cheese types, these bacteria can cause anomalous blowing and color defects, so their presence in milk should be controlled. However, only a few studies considered their distribution in milk and reported that their presence is frequent, ranging between 50% and 95.6%/97% [17,18,19]. Carafa et al. reported that dairy propionibacteria occur more frequently in milk from cows that feed in mountain pastures than in permanent farms [20].

The exploitation of dairy propionibacteria as probiotics, i.e., “live microorganisms that, when administered in adequate amounts, confer a health benefit on the host [21]” started in the 1980s according to a short review published in 1995 on the first findings from in vivo trials. Until then, propionibacteria had been used as growth promoters for farm animals such as calves, piglets, and hens in pure cultures or with lactic acid bacteria. Some *P. freudenreichii* strains promote weight gain, a decreased feed conversion ratio, and improved intestinal health. Though not adhering, *P. freudenreichii* showed a good survival rate in a simulated gastrointestinal transit. Mixed cultures of dairy propionibacteria, bifidobacteria, and lactic acid bacteria showed anticholesterolemic and β-galactosidase activities. Fermented milk containing two strains of *P. freudenreichii* and *Lactobacillus acidophilus* improved the overall health status of elderly people in a small-scale clinical trial. Moreover, dairy propionibacteria stimulated the growth of bifidobacteria and lactobacilli. In addition, they have already been industrially exploited to produce vitamin B12 and propionic acid [22].

Dairy propionibacteria are among the bacterial species involved in dairy technology, exerting health-promoting effects in vivo [23]. According to the latest review article regarding their probiotic properties published in 2017, *P. freudenreichii* and *A. acidipropionici* transiently survive and maintain metabolic activity in the gastrointestinal tract. These two species of dairy propionibacteria promote an increase in intestinal bifidobacteria by producing the bifidogenic molecules 1,4-dihydroxy-2-naphtoic acid (DHNA) and 2-amino-3-carboxy-1,4-naphthoquinone (ACNQ) and also induce a decrease in toxin-producing *Bacteroides* spp. and *Clostridium difficile*. Moreover, selected *P. freudenreichii* strains exerted immunomodulatory and anti-inflammatory effects in human cell lines and mice with induced colitis or mice fed a high-fat diet (HFD). In addition, *P. freudenreichii* favored apoptosis in human cancer cell lines and a mouse tumor model by producing propionate and acetate. The in vivo beneficial effects of *P. freudenreichii* were shown to be mediated by surface layer proteins (Slps) [24].

Currently, *P. freudenreichii* and *A. acidipropionici* are among the microbial species with the qualified presumption of safety (QPS) status for use in food and feed assigned by the European Food Safety Authority (EFSA). Therefore, based on taxonomic identification, the body of knowledge, and the absence of safety concerns, representatives of these species do not have to undergo a complete safety evaluation process and must only satisfy the requirement of not harboring “any acquired antimicrobial resistance genes to clinically relevant antimicrobials” before use in food and feed [25]. Moreover, the species *P. freudenreichii*, *A. acidipropionici*, *A. jensenii*, and *A. thoenii* are included in the list of microbial species with demonstrated safety according to the International Dairy Federation (FIL-IDF) [26].

This descriptive review summarizes the state-of-the-art knowledge on the beneficial effects of dairy propionibacteria on disease prevention and mitigation and their molecular bases to obtain a complete and up-to-date overview of their probiotic potential. To this end, scientific articles regarding the evaluation of their probiotic properties were sought in Google Scholar (https://scholar.google.com/schhp?hl=it, accessed on 25 January 2025) and Embase (https://www-embase-com.bibliosan.idm.oclc.org/, accessed on 25 January 2025) via relevance sorting with the search strings “*Propionibacterium* probiotic” and “*Acidipropionibacterium* probiotic”. In Google Scholar, 80 pages were screened until no further items relevant to the study were retrieved. In Embase, 705 sources were retrieved. The articles finally included in this review were selected after eliminating the duplicates and screening the abstract for content and the full text for originality and novelty. Among articles regarding probiotic mixtures, only those defining the contribution of propionibacteria were considered.

## 2. Molecular Traits of Dairy Propionibacteria Relevant to Probiotic Activities

### 2.1. Genome Features of Dairy Propionibacteria

Dairy propionibacteria are understudied, possibly due to technical difficulties in their isolation, requiring an incubation time of up to seven days using poorly selective or incompletely inclusive media [17,19]. Moreover, their genetic characterization is complicated by their G+C genome content, which is higher than 67% [27]. Indeed, in polymerase chain reaction (PCR) tests and sequencing from these bacteria, additional reagents are needed to facilitate strand and secondary structure dissociation [28]. Whole-genome sequences of dairy propionibacteria recently became available. The complete and annotated genomes available in GenBank are 125, 19, 11, and 1 [https://www.ncbi.nlm.nih.gov/nuccore, accessed on 24 January 2025] for *P. freudenreichii*, *A. jensenii*, *A. acidipropionici*, and *A. thoenii*, respectively.

A recent study used three strains of *P. freudenreichii*, TL19, TL29, and TL110, as representative of high-G + C organisms, to evaluate the performance of different long-read sequencing methods in providing complete and correct sequences and highlighted that different sequencing techniques and assembly methods provided different genome dimensions and numbers of coding sequences (CDSs), even for the same strain. Therefore, appropriate sequencing strategies were defined to obtain optimum completeness and contiguity, correct assemblies, and correct gene content determination. The genome sizes obtained in the study for *P. freudenreichii* ranged between 2,497,808 bp and 2,566,603 bp, and the CDS number ranged between 2158 and 3236 [27].

The most extensive genome sequencing and comparison study on dairy propionibacteria included 20 strains of *P. freudenreichii* isolated from milk, barley, and cheese and applied the long-read sequencing platform PacBio RS II. Eight strains showed genome duplication driven by transposable elements. These transposable elements, ICEs, prophages, and the number of accessory genes varied among the strains. Unique genes were identified. Inversions and rearrangements were observed, but the level of genome co-linearity was high. The composition of the accessory genome highlighted variability in probiotic properties, stress tolerance, phage resistance, and safety. A pilus locus was among the traits encoded by genomic islands. Some of these regions were probably acquired by HGT from *A. acidipropionici*. Putative plasmids were only detected in two strains but lacked a replication origin. Among the S-layer protein genes, known to be involved in the anti-inflammatory properties of *P. freudenreichii*, *slp*A and another *slp* gene were present in all strains, but on genomic islands in some of them. The S-layer protein precursor *ctc*, *slp*E, and *slp*B were present in 13, 12, and 2 strains, respectively [2].

All the strains harbored the gene cluster for the production of vitamin B12, which comprises 33 genes, and, in one strain, some genes of the cluster showed mutations. The three riboswitches that regulate this gene cluster were also conserved. Pilus genes encoding putative subunits of fimbriae anchored to the cell surface, fimbriae major subunits, and a putative sortase C were found in all strains. However, the number of genes and the gene sequences varied, and only one strain showed pilus appendages in transmission electron microscopy (TEM) observation and exhibited mucus-binding capacity [2].

### 2.2. Production of Exopolysaccharides

The production of exopolysaccharides (EPS), which is commonly considered a trait involved in adhesion and immunomodulation [29], was analyzed in 68 *P. freudenreichii* strains and found to vary. A glucosyltransferase *gtf* gene homologous to the *ttf* gene responsible for EPS production in *Streptococcus pneumoniae* was detected via PCR in all the strains. However, only 24 strains agglutinated when exposed to an antiserum specific for the *P. freudenreichii* EPS. Optical microscopy and TEM showed that a capsule was present in some of the agglutinating strains. The Gtf protein was highly conserved among strains, with changes only in six amino acid positions. Its disruption in the *P. freudenreichii*-type strain hindered EPS production, and its heterologous introduction in *Lactococcus lactis* IL1403 conferred the capacity for polysaccharide production, thus showing that *gtf* is the only gene required for EPS production in *P. freudenreichii*. Quantitative reverse transcriptase polymerase chain reaction (qRT-PCR) showed that different levels of EPS production were correlated with the *gtf* transcription level. This was 18 to 264-fold higher in agglutinating strains. At least 107/100 *gtf* transcript copies per ng of RNA were detected in strains with the capsular phenotype. The presence of two putative transposase genes upstream of the *gtf* coding region could be responsible for the overexpression of *gtf* [30].

The presence of a capsule in *P. freudenreichii* was found to hinder its immunomodulation effects by masking the surface molecules responsible for the immune response. About 30% of the strains of this species produce EPS constituted by (1→3,1→2)-β-D-glucan, and strains with an inactivated *gtf*F gene showed a better biofilm formation capacity than the wild type. However, adhesion to Caco-2 cells was not enhanced. Moreover, the induction of anti-inflammatory interleukin IL-10 production in Caco-2 cells was enhanced in Δ*gtf*F mutants compared to the wild type. The presence of the EPS did not increase the survival of *P. freudenreichii* in the mouse gastrointestinal tract [31].

### 2.3. Cell Surface Proteins

The cell surface proteins obtained via guanidine hydrochloride extraction from *P. freudenreichii* CIRM BIA 129 (ITG P20) included internalin A (InlA); the proteins SlpA, SlpB, and SlpE containing the surface layer homology (SLH) domain for anchorage to the cell wall; the large surface protein A LspA; proteins involved in adhesion (BopA), penicillin binding, solute binding, and resuscitation (RpfB factor); and cytoplasmic proteins such as heat shock proteins and translation/elongation factors. Among these, SlpB was the most abundant protein, and its molecular mass was experimentally determined to be 54,147 Da. Shaving with trypsin retrieved a subset of the proteins non-covalently bound to the cell surface, comprising the S-layer proteins. The surface protein extract induced a dose-dependent release of the immunomodulatory cytokines IL-10 and IL-6 by peripheral blood mononuclear cells (PBMCs) [32].

One-dimensional electrophoresis (1-DE) of proteins secreted by 27 strains of *P. freudenreichii* and *A. acidipropionici* showed different patterns for strains of dairy and cereal origins. A 27 kDa transglycosylase was common between the two genera, but with variability at the strain level. Moreover, SlpA, RpfB, and a peptidase of the NlpC/P60 family were the most abundant secreted proteins. In addition, the strains differed in the stress response proteins and central metabolism enzymes detected. Two-dimensional electrophoresis (2-DE) followed by fluorescent staining enabled the detection of InlA, a metallo-endopeptidase M23B, and two proteins more abundant in the dairy strains, namely, a d-alanyl-d-alanine carboxypeptidase, which is involved in peptidoglycan synthesis, and lipoprotein OppA, which is involved in oligopeptide transport. A fimbrillin subunit, FimB, possibly involved in adhesion, was detected in the cereal strain *P. freudenreichii* JS14, which adhered to a hydrophobic material and bovine serum albumin (BSA). None of the strains adhered to mucus [33].

Among the surface proteins in *P. freudenreichii* JS14, SlpB and the aminopeptidase N (PepN) were specifically identified [33]. The gene encoding the latter protein, present in all the dairy propionibacteria-type strains, is present in two paralogs in *A. microaerophilum* [34] and in *A. acidipropionici*, based on the genome sequences [https://www.ncbi.nlm.nih.gov/nuccore, accessed on 20 February 2025]. For the dairy strain *P. freudenreichii* JS22, a putative arabinose isomerase AraI, the Clp family ATPase ClpB, and LspA predominated among the secreted proteins [33].

A *P. freudenreichii* CIRM BIA 129 *slp*B deletion mutant (Δ*slp*B) showed reduced cell surface electronegativity and reduced hydrophobicity, determined as adhesion to hydrocarbon solvents xylol, chloroform, and ethyl acetate. Moreover, the Δ*slp*B mutant was less tolerant to acidic pH, bile salts, and heat stress. In this mutant strain, an exported enolase involved in immunomodulation and adhesion, SlpA, SlpD, SlpE, and InlA, were downregulated [35].

The anti-inflammatory properties of *P. freudenreichii* were initially demonstrated for the strain JS. This strain reduced the expression of the pro-inflammatory interleukin IL-8 in Caco-2 cells infected with *Helicobacter pilori* and increased the expression of the anti-inflammatory cytokine IL-10 in PBMC. Among 23 *P. freudenreichii* strains, only *P. freudenreichii* CIRM BIA 129 induced this cytokine at levels comparable to the anti-inflammatory probiotic *Bifidobacterium longum* BB536. The pro-inflammatory cytokines interferon γ (IFN-γ) and tumor necrosis factor α (TNF-α) were induced very weakly, while interleukin IL-12 was not expressed. Guanidine hydrochloride extracts contained 174 proteins unique to single strains. SlpA, SlpB, SlpE, LpsA, and a protein with a glucosaminidase domain were expressed in one or more strains [31].

Genome, transcriptome, and surface proteome analysis in *P. freudenreichii* strains indicated that 158, 161, and 11 genes, respectively, were positively associated with the induction of IL-10, and SlpE was identified in all three datasets. Among fifteen genes with the strongest positive or negative association with IL-10 induction, *slpB*, *slpE*, *slpF*, *hsdM3*, *pep*, *htrA4*, *eno1*, and *pouf* were successfully inactivated in different strains. The inactivation of *slpB*, *slpE*, and *hsdM3* in *P. freudenreichii* CIRM BIA 129 caused a remarkably lower induction of IL-10 in PBMCs; the inactivation of *slpB*, *slpF*, and *pouf 10925* reduced the secretion of tumor necrosis factor TNF-α and IFN-γ; and the inactivation of *slpE* reduced the secretion of TNF-α. The inactivation of *eno1*, *htrA4*, and *pouf 08235* in the weakly anti-inflammatory strain CIRM BIA 121 led to the increased induction of IL-10, TNF-α, and IL-6 [36].

The SlpB protein is also involved in the adhesion of *P. freudenreichii* to human intestinal epithelial cells HT29. Different amounts of this protein were detected in four of seven *P. freudenreichii* strains tested. *P. freudenreichii* CIRM BIA 129 showed the highest adhesion rate. Strain CIRM BIA 118, which also expressed SlpB, exhibited about half its adhesion rate. The strain with the lowest adhesion rate did not express S-layer proteins. The *P. freudenreichii* strains were not internalized in the intestinal cells, and scanning electron microscopy highlighted their localization on the brush border. The adhesion capacity was lost after shaving with trypsin and after deleting the *slp*B gene [37]. *P. freudenreichii* CIRM BIA 129 or its S-layer proteins attenuated the upregulation of the pro-inflammatory IL-8 and IL-12 interleukins and TNF-α in HT29 cells exposed to the *Escherichia coli* lipopolysaccharide (LPS). Moreover, *P. freudenreichii* induced the upregulation of IL-10 both in the presence and absence of LPS, and its Slps stimulated an increase in tumor growth factor β (TGF-β) [38].

A Δ*slp*B mutant of *P. freudenreichii* CIRM BIA 129 showed decreased adhesion to the intestinal epithelial barrier (IEB) in in vitro models composed of Caco-2 cell monolayers. Moreover, differing from the wild type, it did not reduce trans-epithelial electrical resistance (TEER), and it did not prevent paracellular permeability to sulfonic acid-fluorescein isothiocyanate (FITC) and transcellular permeability to horse radish peroxidase (HRP) after the induction of inflammation. Western blotting highlighted that the Δ*slp*B mutant did not prevent a decrease in the zonula occludens 1 (ZO-1) tight junction protein expression. This protein and Claudin-2 decrease in the case of IBDs such as ulcerative colitis (UC) and Crohn’s disease (CD). The consequently increased gut permeability leads to inflammation, with further damage induced by IFN-γ and TNF-α [39]. The organic acids produced by *P. freudenreichii* CIRM BIA 129 and its Δ*slp*B mutant did not significantly influence the inflammation-induced permeability, thus proving the essential role of SlpB [39].

### 2.4. Extracellular Vesicles

The cells of most organisms produce extracellular vesicles (EVs) that are nanoparticles delimited by a lipid bilayer membrane and contain proteins, nucleic acids, and metabolites. EVs are involved in the transfer of virulence and antibiotic resistance genes and the interaction of probiotic microorganisms with host cells. Beneficial effects were observed for the EVs produced by the probiotic bacteria *Lacticaseibacillus paracasei* BL23, *L. rhamnosus* GG, *Lactiplantibacillus plantarum* WCFS1, and *B. longum* KACC 91563. The EVs produced in ultrafiltered (UF) milk by *P. freudenreichii* CIRM BIA 129 had a diameter of about 84 nm and contained 319 proteins, representing 11% of the whole cell proteome, including proteins involved in all cell functions and proteins involved in immunomodulation, such as the enolase EnoI, the internalin InlA, the aconitase Acn, the glutamine synthetase Gln1, the glucose-6-phosphate isomerase Gpi, the triosephosphate isomerase Tpi, SlpB, and the chaperonin GroEL [40].

The in silico prediction based on machine learning (interSPPI, http://zzdlab.com/intersppi/index.php, accessed on 30 March 2025) and the homology (interolog) of protein–protein interactions for *P. freudenreichii* EV-associated proteins highlighted interactions with human proteins involved in metabolism, signal transduction, infectious diseases, and immunity. Among these, the p105 subunit of the nuclear factor kappa-light-chain-enhancer of activated B cells (NF-κB) and members of its signaling pathway, such as adapters and Toll-like receptors, showed the highest number of interactions. A dose-dependent effect of *P. freudenreichii* EVs on reducing NF-κB activation was demonstrated in HT29/kb-seap-25 cells exposed to *Escherichia coli* LPS but not in those treated with the inducers of inflammation, TNF-α and IL-1β. In addition, *P. freudenreichii* EVs reduced cytokine IL-8 dose-dependently [40].

EVs produced in UF milk reduced the regulatory activity of NF-kB more efficiently than those formed in yeast extract lactate medium (YEL) by *P. freudenreichii* CIRM BIA 129 in HT29 cells with *E. coli* LPS-induced inflammation. The two EV types shared 358 proteins, and those from UF milk also contained proteins involved in carbohydrate and amino acid metabolism, as well as DNA processing [41]. Other identified proteins were involved in the production of secondary metabolites; metabolism in different environments; oxidative phosphorylation; peptidoglycan synthesis; ribosome composition; protein export; quorum sensing (QS); and immunomodulation, including SlpB, SlpE, EnoI, Acn, GroEL2, and a membrane-associated hypothetical lipoprotein [42]. EVs produced by *P. freudenreichii* CIRM BIA 129 contain a correctly conformed SlpB protein, and those purified via size-exclusion chromatography inhibited an increase in paracellular permeability to sulfonic acid-FITC in a cell monolayer, thus confirming the role of SlpB in mitigating inflammation-induced changes [39].

## 3. Beneficial In Vivo Effects of Dairy Propionibacteria

Dairy propionibacteria positively affected animal models of conditions such as intestinal inflammation, colorectal cancer (CRC), bone diseases, and infections. The best-studied species was *P. freudenreichii*, for which different strains were tested in vivo and characterized in terms of the molecular mechanisms of action [14,43,44,45,46,47,48,49,50,51,52,53,54,55,56,57,58,59,60,61,62,63,64,65,66,67,68,69,70].

The beneficial effects exerted in vivo are possible due to the ability of dairy propionibacteria to survive in the gastrointestinal tract (GIT) during transit. Indeed, it was reported that *P. freudenreichii* CIRM BIA 129 supplied to piglets at a level of 11 Log CFU in culture or cheese was retrieved at about 7 Log CFU/g in feces [8]. *A. jensenii* 702 was fed to rats and retrieved from feces in high numbers [71]. The strain *P. freudenreichii* JS was recovered in high numbers from the feces of healthy subjects who consumed fruit juice enriched with whey containing this bacterium. However, gut colonization was transient [72]. Trials of growth promotion in farm animals highlighted the capacity of dairy propionibacteria to maintain viability in GIT [73]. *A. jensenii* 702 used as a direct-fed microbial (DFM) in calves was recovered from feces at about 5 Log CFU/g during the treatment period when administered in daily doses of 9 Log CFU/kg [74]. The same strain was supplied to layer chickens in doses of 7 Log CFU per day and was proven to survive transit in the GIT [75].

It was observed that *P. freudenreichii* CIRM BIA 129 inglobated in a cheese matrix did not lose viability during the gastric and small intestine phases in the in vitro dynamic DIDGI^®^ digestion system and remained viable at high levels until the end of the process. Separation on sodium dodecyl sulfate–polyacrylamide gel (SDS-PAGE) and Western blotting showed that after in vitro static digestion, SlpB remained intact if *P. freudenreichii* was supplied in cheese. However, it was degraded after the gastric phase when the strain was supplied in UF milk. In the dynamic digestion system, SlpB remained intact in the duodenum phase when supplied with cheese for longer than when supplied with UF milk; thus, cheese better preserves its probiotic properties [38].

The adhesion capacity of dairy propionibacteria to intestinal cells was evaluated on normal ileal mucosa cells, namely, IPEC-J2 cells, and it was shown that the type strains of *P. freudenreichii*, *A. acidipropionici*, *A. jensenii*, and *A. thoenii* adhered at rates comparable to *L. reuteri* 12002 used as a positive control. *P. freudenreichii* showed the highest adhesion percentages, which were enhanced in the presence of calcium [73].

The dairy propionibacteria showed tolerance to low pH values, and none of them significantly declined after 3 h at pH 2.5. Moreover, the *A. acidipropionici* strain showed a negligible decrease after 24 h, while the other species lost about 30% viability [76]. However, the data in the literature are not concordant regarding the tolerance of dairy propionibacteria to a low pH, thus indicating strain-to-strain variability [77]. The same is true for tolerance to bile salts, for which high tolerance or high sensitivity was reported for the 0.3% (*w*/*v*) concentration. The discordance could be explained by variability in testing conditions [76]. Ibrahim et al. [77] reported little viability loss for some strains exposed to 1% bile salts for 48 h.

### 3.1. Prevention and Mitigation of Intestinal Inflammatory Diseases

Inflammatory bowel diseases (IBDs) are characterized by intestinal dysbiosis, i.e., a less diverse and more unstable intestinal microbiota than that found in healthy individuals and a decrease in the microbial components with an immunomodulatory role [49]. Probiotics help reinstate microbial groups with beneficial roles and decrease detrimental microorganisms. Among dairy propionibacteria, *A. jensenii* 702 has been used in mixed commercial probiotic preparations due to its ability to support an increase in bifidobacteria [78]. An in vitro study showed that *P. freudenreichii* W200 (Winclove Probiotics, Amsterdam, The Netherlands) was the only probiotic that did not induce an increase in *Bacteroides-Prevotella* spp. in fecal cultures and induced a higher production of lactate among the SCFAs compared to probiotic strains belonging to the species *Heyndrickxia coagulans*, *Bacillus subtilis*, *Levilactobacillus reuteri*, and *L. rhamnosus* [79].

Most studies on the role of dairy propionibacteria in mitigating inflammatory intestinal diseases evaluated their effects on immunomodulation and intestinal tissue health. Propionate was the first metabolite from dairy propionibacteria proven to alleviate colitis induced by 2,4,6-trinitrobenzenesulfonic acid (TNBS) in rats receiving whey cultures fermented by the Swiss-type cheese isolate *P. freudenreichii* ET-3 [43]. In murine colitis induced with sodium dextran sulfate (DSS), the *P. freudenreichii* DHNA metabolite attenuated the expression of mucosal addressin cell adhesion molecule 1 (MAdCAM-1), which favors leucocyte infiltration. It was linked to a lower colon mucosa damage score, a reduction in the number of β7 integrin-positive cells that mediate the adhesion of leucocytes to vascular endothelial cells; significant suppression of IL-1β, IL-6, and TNF-α mRNA levels; and a substantial increase in the number of lactobacilli and amounts of SCFAs [44].

The mechanism of colitis inhibition observed for *P. freudenreichii* ET-3 involves the activation of the aryl hydrocarbon receptor (AhR) transcriptional factor that activates genes with a xenobiotic-responsive element (XRE) consensus sequence in the promoter and is involved in xenobiotic detoxification. AhR-regulated pathways, which can be activated in the gut by some probiotic bacteria, suppress IBDs. Among metabolites produced by *P. freudenreichii* ET-3, DHNA increased the transcription level of cytochrome P450 family 1 subfamily A member 1 (CYP1A1) induced by AhR in Caco2 cells without affecting their viability. This effect also occurred in mice, mainly in the upper intestine [45]. Moreover, DHNA increased Ahr-induced anti-microbial C-type lectins RegIII, which inhibits DSS-induced colitis in mice. DHNA also inhibited the production of IL-6 in bone marrow macrophages upon exposure to LPS without showing toxicity for these cells, so it could represent a nontoxic Ahr activator for colitis prevention [45].

The most abundant components of the surface proteome of *P. freudenreichii*, CIRM BIA 129, grown in a cheese comprising only this strain and identified using MS/MS, were SlpA, SlpB, SlpE, InlA, and the chaperonins Hsp20, GroEL1, and GroEL2 that are involved in immunomodulation. The administration of this cheese to mice with TNBS-induced colitis significantly prevented weight loss and reduced disease scores and the inflammation marker IL-6 in blood. The attenuation of colon inflammation was indicated by the increased expression of *Pparγ*, which encodes the peroxisome proliferator-activated receptor γ. The attenuation of TNBS-induced oxidative stress in the colon was inferred from the decreased expression of the gene *cox2*, which encodes cytochrome c oxidase subunit 2. Moreover, the restoration of the intestinal barrier was indicated by the increased expression of the *Zo*1 gene for the zonula occludens 1 protein [46].

Similar effects were observed when *P. freudenreichii* CIRM BIA 129 was supplied to mice with TNBS-induced colitis in a cheese co-fermented with *Lactobacillus delbrueckii* subsp. *lactis*. In this case, a decrease in the oxidation marker heme oxygenase 1 (*Hmox*) also occurred, and *P. freudenreichii* enhanced the protective effect of the lactobacilli by reducing weight loss and inflammatory signs. An increase in the Rikenellaceae intestinal microbiota component, considered a consequence of inhibiting the inflammatory response mediated by NF-kB, was observed [47].

*P. freudenreichii* CIRM BIA 129 reached levels of 9/10 Log CFU/g in an experimental cheese in which it was added alone and in industrial Emmental cheese in which it was added together with *Streptococcus thermophilus* and *L. delbrueckii*. Both *P. freudenreichii* cheeses mitigated weight loss and the disease activity index (DAI) in mice with DSS-induced colitis. Moreover, the industrial Emmental cheese reduced mucosa alterations and cell infiltration, increased the expression of ZO-1, and reduced the levels of IL-6 and IL-17. Both *P. freudenreichii* cheeses decreased immunoglobulin IgA production in the small intestine, indicating reduced disturbance of the intestinal barrier [48].

*P. freudenreichii* CIRM BIA 129 was used to ferment UF skim or whole milk administered to mice with DSS-induced colitis. Both types of milk fermented by *P. freudenreichii* alleviated colon shortening, a decrease in goblet cells, and crypt length reduction. However, only the fermented whole milk kept the disease score significantly below that of the positive control. In addition, milk fermented by *P. freudenreichii* reduced the gut permeability, measured as the transfer of radiolabeled diethylenetriamine pentaacetic acid (DTPA) in blood. The fermented whole milk decreased pro-inflammatory *il1b* and *il*6 and nitric oxide synthase 2 *nos*2 gene expression, as well as the upregulation of *Pparγ*, more than skim milk [49].

Milk ultrafiltrate (MUF) fermented by *P. freudenreichii* CIRM BIA 129, administered to mice before colitis induction with DSS, decreased disease indexes, bleeding, and stool consistency but not weight loss. Gut permeability, measured by administering sulfonic acid-FITC and determining its concentration in plasma, was significantly lower in the fermented MUF group. Intracellular permeability, measured as the activity of administered HRP in plasma, was also reduced. Histopathological observation showed that in the *P. freudenreichii* group, the crypt length did not decrease [39].

The intestinal mucus layer is a physical barrier against pathogens and harmful chemicals and protects against IBD. The *P. freudenreichii* KCTC 1063 culture supernatant at a 10% (v/v) concentration, containing the SCFAs propionate, acetate, and butyrate, was not cytotoxic for LS 174T goblet cells and stimulated the expression of MUC2, the most abundant gel-forming mucin in the human intestine, which is a glycosylated *O*-glycoprotein [50]. When administered to rats with DSS-induced colitis, *P. freudenreichii* KCTC 1063 and its culture supernatant resulted in a histological disease score equal to that of the negative control and a less damaged crypt structure, with a lower leucocyte infiltration degree, respectively. Both groups treated with *P. freudenreichii* showed a goblet cell number similar to that of the healthy control. MUC2 expression was either not significantly different from or higher than that in the healthy control for live *P. freudenreichii* and the culture supernatant, respectively. After an initial decrease, body weight increased rapidly in the living *P. freudenreichii* and culture supernatant-treated groups. These groups exhibited less severe diarrhea and bleeding; a normal colon length; lower levels of *TNF-α*, *IL-6*, and *IL-1β* in the distal colon; and higher fecal contents of propionate and butyrate SCFAs. *P. freudenreichii* was administered for seven days at 8 Log CFU and remained at 5 Log CFU/g of feces until the end of the experiment on day 29, thus indicating gut colonization [50].

In UC, harmful bacteria exceed beneficial bacteria in the intestinal microbiota, and equilibrium can be restored by supplying probiotics. *P. freudenreichii* B1 was compared with *B. bifidum* H3-R2 and *C. butyricum* C1-6 to determine the effects on DSS-induced colitis in mice. Like the other two probiotics, this strain prevented colon shortening and decreased the DAI; decreased pro-inflammatory cytokines IL-8, IL-1β, and TNF-α; and increased IL-10. Moreover, it decreased Toll-like receptors TLRs 2 and 4 and increased TLR-5; enhanced the expression of ZO-1 and claudin-1 similarly to *C. butyricum*; and increased the expression of R-spondin-3 (Rspo3), which is active in the repair of damaged tissues. *P. freudenreichii* and *B. bifidum* more efficiently downregulated RHO kinase ROCK-1 and Axin2, which inactivate the Wnt/β-catenin pathway responsible for regenerating the intestinal epithelium. *P. freudenreichii* and *C. butyricum* better preserved the diversity of the intestinal microbiota, which did not significantly differ from that of the positive control group gavaged daily with mesalazine (5-AS). Lactobacilli and bifidobacteria were enriched in all the probiotic groups, but *P. freudenreichii* induced higher increases in SCFAs that negatively correlated with *Escherichia*-*Shigella*, *Staphylococcus*, and *Enterobacter* spp. levels and pro-inflammatory indexes and positively correlated with anti-inflammatory molecules such as IL-10 and TLR-5 [51].

Mucositis is a severe inflammation of the small bowel in cancer patients treated with radiotherapy or chemotherapy and is characterized by degenerated enterocytes and goblet cells; the infiltration of leucocytes in the lamina propria; and the increased production of mucus, atrophic villi, and hypoplastic crypts. Symptoms include diarrhea and weight loss, which are treated with antimicrobials and anti-inflammatory agents. This treatment may be ineffective or not well tolerated. Therefore, treatment with probiotics should be considered as an alternative. *P. freudenreichii* CIRM BIA 129 and its ΔSlpB mutant were evaluated to determine their effects on mucositis in vitro and in mice with mucositis induced by the chemotherapy drug 5-fluorouracile (5-FU). In HT29 cells exposed to *E. coli* LPS, the wild-type strain and the purified SlpB induced IL-10 and inhibited IL-8, *ifnγ*, and *tfna* expression. Moreover, the *P. freudenreichii* wild type induced *tlr2* and repressed *tlr4* and *tlr*9 [50]. In mice with induced mucositis, the *P. freudenreichii* wild type significantly reduced weight loss, leucocyte infiltration, and ulceration in the intestinal mucosa, partially restoring the height of villi and the granular density of Paneth cells. This strain reduced the gut permeability induced by 5-FU and expressed as the extraintestinal translocation of radiolabeled 99mTc-DTPA by increasing claudin-1 *cld1* gene expression. Moreover, the expression of IL-17a, IL-12, and IL-1β in the intestinal mucosa was reduced. These effects were not observed for the Δ*slp*B mutant strains [52].

Newborns are particularly susceptible to infections because their immune system is immature, and the number of protective T cells is insufficient. Preterm neonates are exposed to the risk of necrotizing enterocolitis (NEC) caused by the deterioration of the intestinal microbiota and inflammation induced by maternal pathogens. Establishing a protective intestinal microbiota supplied by breast milk stimulates the development of an immune system that contrasts enteric pathogens and reduces the risk of NEC [53].

A metagenome analysis of the intestinal microbiota of 20 preterm newborns receiving breast milk and 20 preterm newborns receiving formula milk showed that the first group harbored a more diverse and balanced microbial population comprising actinobacteria, among which *P. freudenreichii* predominated and increased over time. A strain sharing the highest genome identity with *P. freudenreichii* DSM 20271 and denominated *Propionibacterium* P. UF1 was isolated from the feces of a breast-fed infant and transiently colonized the gut for six days in C57BL/6 mice and longer in germ-free mice [14].

Similarly to the intestinal microbiota of breast-fed newborns transferred to mice, *P. freudenreichii* P.UF1 did not increase IL-1β in colon dendritic cells (DCs), while the microbiota from formula-fed newborns induced higher levels of this pro-inflammatory cytokine. In addition, T helper 17 (Th17) cells—particularly IL-10+IFN-γ–, as well as anti-inflammatory regulatory T cells (Tregs) expressing IL-10 (IL-10+ Tregs)—increased, while the level of γ-proteobacteria decreased [14].

Dihydrolipoamide acetyltransferase (DlaT), a cytoplasmic enzyme of the pyruvate decarboxylase complex, was the main component of the guanidine hydrochloride surface protein extracts of *P. freudenreichii* P.UF1 and was able to induce IL-10+ Th17 cells. The differentiation of Th17 cells from CD4+T cells induced by *P. freudenreichii* was mediated by major histocompatibility complex II (MHC II) since it did not occur in *MHC II*--/-- mice. A Δ*dlaT* mutant of *P. freudenreichii* P.UF1 did not induce the differentiation of IL-10+ Th17 cells in the mouse colon. Complementation of this mutant with three DlaT peptides restored the differentiation of Th17 cells and the induction of IL-10+ Tregs. The C-type lectin receptor (CLR) SIGNR1, which recognizes bacteria from their surface composition and stimulates DCs to induce T cell differentiation, was upregulated in mice receiving *P. freudenreichii* P.UF1. Its involvement in the attenuation of inflammation was demonstrated by showing that in *Signr1*--/-- mice infected with a *Listeria monocytogenes* mutant incapable of systemic spread, the differentiation of Th17 cells and the induction of IL-10+ Tregs did not occur, while they occurred in *Signr*+/+ mice [14].

The colon microbiota of germ-free mice gavaged with *P. freudenreichii* P.UF1 was enriched with lactobacilli and *Ruminococcus* spp., which showed its ability to modify the intestinal microbiota composition. Since the intestinal microbiota of the mother influences the immune system of the newborn, *P. freudenreichii* P.UF1 was administered to pregnant mice. The newborns from these mothers exhibited an increase in DCs expressing the transforming growth factor TGF-β and IL-10, as well as an increase in IL-10+Th17 and IL-10+ Tregs. In addition, the survival rate increased in newborns submitted to NEC-like injury induced via gavage with a mixture of commensal bacteria from adults and exposure to hypoxia. In these newborns, the transcription of the inflammatory nitric oxide synthase *iNOS* and interleukins *Il-1b*, *Il-6*, and *Il-23* was reduced. Moreover, innate lymphoid cells 3 (ILC3) expressing IL-17A and IL-22, which protect from bacterial infections and mediate tissue repair, increased in the small intestine [14].

In a murine systemic infection with *L. monocytogenes* 10403S, a strain with a mutation in *inl*A used to control the infection kinetics, *P. freudenreichii* P.UF1, differently from its Δ*dlaT* mutant, reduced IL-1β, IL-6, and IL-12/IL23p40 produced by DCs and reduced Th1 cells producing IFNγ. Moreover, it increased Th17 cells, IL-10+ Treg cells, intestinal bifidogenic bacteria, and the clearance of *L. monocytogenes* from feces and tissues, showing both local and peripheral inflammation mitigation. Three DlaT peptides were cloned in the *L. monocytogenes* strain, and the infection of mice with this strain induced similar immunomodulatory effects as those observed in mice receiving *P. freudenreichii* P.UF1, thus confirming the immunomodulatory role of DlaT [14].

The Th17 cells induced by *P. freudenreichii* P.UF1 were responsible for mitigating *L. monocytogenes* infection, as demonstrated by neutralizing the IL-17A produced by these cells with specific antibodies injected in mice. In mice defective in the recombination-activating gene required for generating T and B cells, the pathogen also persisted in the presence of *P. freudenreichii* P.UF1. In Th17 cells induced by *P. freudenreichii*, the upregulation of pathways responsible for producing extracellular matrix components involved in tissue healing and genes involved in immune regulation was observed via transcriptome analysis. Finally, higher numbers of Lactobacillaceae and Clostridiaceae able to produce SCFAs and lower levels of *Prevotella* spp. in the intestinal microbiota; significantly increased levels of vitamins B2, B5, and B9; and decreased pro-inflammatory molecules prostaglandine E1 and 20-Hydroxyleukotriene E4 were observed in mice receiving *P. freudenreichii* P.UF1 [53].

The in vivo effects exerted by *P. freudenreichii* strains in intestinal inflammation models are summarized in Table 1.

### 3.2. Immunomodulation

*P. freudenreichii* CIRM BIA 129 was selected among ten strains to determine the anti-inflammatory effects in vitro. UF milk fermented by this strain supplied to piglets improved food intake and the growth rate. Moreover, it decreased IL-8 and TNF-α in the colon mucosa and increased bifidobacteria in feces [8]. After 14 days of treatment, total SCFAs and branched-chain fatty acids (BCFAs) were significantly higher in the animals receiving *P. freudenreichii*. Slps from this bacterium increased IL-10 and reduced TNFα and IFNγ production in piglet PBMCs and mesenteric lymph node immune cells (MLNCs) in which inflammation was induced with *E. coli* LPS or concanavalin A. In MLNCs, *P. freudenreichii* supplied with cheese enhanced the expression of the regulators GATA binding protein 3 (GATA3) and forkhead box P3 (FoxP3) [80] involved in immune tolerance [81].

Regardless of the method of administration, *P. freudenreichii* CIRM BIA 129 increased the ratio between anti-inflammatory Treg and pro-inflammatory Th17 cells. Ex vivo stimulation with LPS of PBMC from piglets treated with *P. freudenreichii* showed an increased secretion of IL-10, while stimulation with concanavalin A increased IFNγ secretion in MLNCs from the group treated with *P. freudenreichii* supplied in cheese. Importantly, IFNγ induced the intestinal immune response against pathogens via macrophage activation and caused an increase in Th1 cells [80]. The same strain was used to ferment sweet whey and transform a by-product into a functional food. When this whey was supplied to piglets for 14 days, a significant increase in fecal bifidobacteria occurred. MLNCs isolated after fermented whey administration showed an upregulation of T-box expressed in T cells (T-bet), a regulator of the Th1 response [82].

*A. jensenii* 702 isolated from raw cow milk in Australia was tested in rats as an adjuvant for an oral vaccine constituted by a culture filtrate of *Mycobacterium tuberculosis* H37Rv. The *A. jensenii* strain survived the passage through the gastrointestinal tract and was recovered from feces throughout the entire trial. This strain activated spleen T cells more than the cholera toxin, a strong mucosal adjuvant. The IFNγ levels, 2 to 3 Log higher than IL-4 in the *A. jensenii* groups, indicated a Th1 response that is effective in protecting against tuberculosis [54].

The *Caenorhabditis elegans* worm is an animal model used to study the effects of different agents on innate immunity and aging. Feeding *C. elegans* on a cell lawn of *P. freudenreichii* KCTC 1063 prolonged its lifespan by 13% compared to conventional feeding on *E. coli* OP50; increased its body movements, which indicate muscular strength; and decreased the accumulation of lipid granules, indicators of aging. The immunity-related pathways of the DNA binding ligand (DBL)/TGF-β; P38 mitogen-activated protein kinase (MAPK), involved in innate immunity; Daf-2/DAF-16, involved in the response to insulin/insulin-like growth factor-1 (IGF-1) signaling (IIS); and the *lys-7* and *lys-8* genes for antimicrobial peptides were upregulated. Comparison with a *C. elegans* mutant defective in innate immunity and lifespan extension-related genes highlighted that *P. freudenreichii* regulates the p38 MAPK and TGF-β pathways. Activating the p38 MAPK pathway in *C. elegans* increased resistance to *Salmonella* Typhimurium infection [55].

In mice, *P. freudenreichii* JS increased the number of natural killer (NK) cells in the liver and decreased cells with increased IFN-γ production in the spleen. Moreover, this strain increased IL-6 levels in serum and decreased the Th1/Th2 average ratio in the spleen and the Treg/Th17 ratio in both the liver and spleen. It also increased the percentage of Th17 cells in CD4+T cells in both the liver and spleen and the percentage of Treg cells in CD4+T cells in the spleen, thus showing a pro-inflammatory effect for most parameters [83].

In allergic reactions, Th2 cells are induced and produce cytokines that promote the proliferation of B cells and immunoglobulin class switching to IgE, which stimulate the release of histamine and other inflammatory substances from mast cells and basophils. New exposure to the allergen induces the production of mediators that lead to the proliferation of Th2 cells and the activation of DCs, resulting in a reduction in allergen-specific Treg cells and the activation of mast cells. The intestinal microbiota balances inflammatory reactions and immune tolerance via its action on the innate and adaptive immune responses, as demonstrated with experiments in germ-free mice. SCFAs produced by components of the intestinal microbiota stimulate the production of innate lymphoid cells (ILCs) that are distributed in peripheral tissues and promote homeostasis during inflammation [56].

*P. freudenreichii* CIRM BIA 129, administered at a daily dose of 9 Log CFU to mice with a food allergy induced by oral sensitization with wheat gliadins, prevented an increase in body temperature and gliadin-specific IgE and IgG1 levels in serum. Moreover, it increased gliadin-specific IgG2 levels. The level of the mucosal mast cell protease-1 (mMCP-1) activation marker decreased in this group. The levels of Th2 cells in mesenteric lymph nodes were not significantly different from the control, and the associated cytokines IL-5 and IL-13 were lower than in the food allergy-positive controls. Treg cells, which regulate Th1 and Th2 responses, increased in the group treated with *P. freudenreichii* CIRM BIA 129. Therefore, it was concluded that this bacterial strain exerted an anti-inflammatory effect by limiting the Th2 response. In addition, it prevented ILC2’s increase in Peyer’s patches and significantly reduced intestinal paracellular and transcellular permeability, which was determined by measuring the translocation of sulfonic acid-FITC and HRP, respectively. Indeed, the expression of ZO-1 was unaltered. PBMCs from healthy volunteers co-cultured with *P. freudenreichii* CIRM-BIA 129 showed increased TGF-*β* levels. Furthermore, this strain increased IL-10 and decreased IFN-*γ* production by monocyte-derived DCs (MoDCs). The role of SlpB in these effects was demonstrated by a comparison with the ΔSlpB *P. freudenreichii* CIRM BIA 129 mutant [56].

The potential protective effects of *P. freudenreichii* from infections of the respiratory system were indicated by the ability of strain PF-24 to induce an oxidative burst in bovine alveolar lavage cells (BALs), represented mainly by macrophages. Flow cytometry coupled with fluorescent antibodies showed that this strain also induced a higher percentage of cells expressing CD14 necessary for recognizing Gram-negative bacteria, and an increase, though not significant, of cells expressing the CD205 DC marker that modulates the immune response to microbial infections [84], thus showing a general capacity to enhance leukocyte functions [85].

Table 2 summarizes the in vivo effects exerted by dairy propionibacteria on the immune response.

### 3.3. Effects on Obesity

A high-fat diet (HFD) predisposes individuals to chronic conditions such as obesity, type 2 diabetes, cardiovascular diseases, and fatty liver disease preceded by low-level intestinal inflammation and insulin resistance. ApoE*3Leiden transgenic mice are animal models of this condition that express a mutated human *APOE3* gene associated with human dys-beta-lipoproteinaemia and also harbor a human *APOC1* gene that induces high levels of lipoproteins and triglycerides in plasma. *P. freudenreichii* JS reduced the weight gain and the increase in gonadal adipose tissue in these mice consuming an HFD to a greater extent than *L. rhamnosus* GG. Moreover, in the *P. freudenreichii* group, the adhesion molecule VCAM-1 marker of vascular inflammation in plasma, the mast cell number, and the TNF-α level were lower. Like *L. rhamnosus* GG, *P. freudenreichii* JS decreased alanine aminotransferase (ALT) values, leaving the adiponectin levels unaltered [57].

Preadipocytes 3T3-L1 exposed to differentiation-inducing 3-isobutyl-1-methylxanthine, dexamethasone, and insulin (MDI) and incubated with heat-killed raw milk isolate *P. freudenreichii* MJ2 (hkMJ2) at numbers as high as 8 Log CFU/mL did not lose viability (determined using the 3-[4,5-dimethylthiazol-2-yl]-2,5-diphenyltetrazolium bromide (MTT) assay). It was observed that hkMJ2 reduced fat accumulation in a dose-dependent manner in these cells more efficiently than an anti-obesity *L. plantarum* KACC15357 probiotic. Moreover, hkMJ2 increased the expression of the transmembrane protein preadipocyte factor-1 (Pref-1), which supports the maintenance of the preadipocyte stage, and decreased the expression of genes encoding the adipogenic proteins PPARγ, CCAAT/enhancer-binding protein alpha (C/EBPα), fatty acid synthase (FAS), stearoyl-CoA desaturase-1 (SCD-1), and acetyl-CoA carboxylase (ACC) [58].

Treatment of HFD-induced obese mice with live *P. freudenreichii* MJ2, hkMJ2, and *L. plantarum* KACC15357 decreased the body weight by 31%, 22.8%, and 18.5%, respectively, and decreased the food efficiency ratio. *P. freudenreichii* MJ2 and hkMJ2 decreased the expression of *PPARγ*, *FAS*, *SCD-1*, and *ACC* in epididymal white adipose tissue (eWAT); increased the expression of the lipolytic enzymes adipose tissue triglyceride lipase (ATGL), hormone-sensitive lipase (HSL), and carnitine palmitoyltransferase 1α (CPT-1α); and increased the enzyme involved in β-oxidation of fatty acids peroxisomal acyl-coenzyme A oxidase 1 (ACOX1). Moreover, treatment with *P. freudenreichii* MJ2 and hkMJ2 decreased the size of adipocytes in eWAT [58].

Low-density lipoprotein (LDL) cholesterol in serum significantly decreased in the *P. freudenreichii* MJ2 group, and the ratio of high-density lipoprotein cholesterol (HDL)/total cholesterol (TCHO) increased in all probiotic-treated groups. The fasting glucose levels decreased in the *P. freudenreichii* MJ2 group, and insulin levels decreased for both the MJ2 and hkMJ2 groups, with reduced homeostasis model assessment insulin resistance (HOMA-IR) scores. Staining with hematoxylin and eosin showed significantly reduced lipid accumulation in hepatocytes of these groups and significantly lower levels of serum glutamic oxaloacetic transaminase (GOT) and glutamic pyruvic transaminase (GPT) indexes of hepatic damage [58].

Obesity is caused by a remodeling of the adipose tissue induced by the imbalance between energy intake and expenditure. In this condition, the adipocytes originating from mesenchymal stem cells (MSCs) become hypertrophic and hyperplastic. Molecules that impair this process could help treat the condition. The cell wall (CW) fraction of *P. freudenreichii* MJ2 and hkMJ2 reduced lipid levels in 3T3-L1 preadipocytes in which lipid accumulation was induced with MDI during differentiation. This effect was exerted by the CW fraction and the separated surface protein component (SP), which caused a reduction in the expression level of the lipogenesis factor *Fas*, but not by the CW fraction deprived of the SP fraction [59].

The protein fraction of the SP components with a molecular weight above 35 kDa, separated via Q fast, anion exchange, and size exclusion chromatography, reduced lipid accumulation. Among these proteins, chaperonin 60 (Cpn60), also designated heat shock protein 60 (Hsp60) or GroEL, was expressed as a recombinant protein. When tested on 3T3-L1 cells, it was found to reduce adipogenesis to a dose-dependent extent. This effect was most likely attributable to the decreased expression of genes *Pparγ*, *Cepba*, *Fas*, and *Scd1*. Indeed, the terminal differentiation stage from MSC to adipocytes is mediated by the master regulators PPARγ and C/EBPα that induce the lipogenesis genes FAS and SCD1. The expression levels of the respective genes and the content of lipid droplets progressively decreased for eight days during adipocyte differentiation. Indeed, there was a decrease in PPARγ translocation to the nucleus and its binding to the promoters of the target genes [59].

The level of the C/EBPβ-positive regulator of the genes *Pparγ* and *Cebpa* in the nucleus and its binding to their promoters—determined using Western blotting and chromatin immunoprecipitation (ChIP)—showed a decrease after 24 h of exposure to the *P. freudenreichii* GroEL. In addition, there was an upregulation of *GATA*2 and *GATA*3 genes encoding the suppressors of adipocyte differentiation, which inhibit the expression of *Cebpa* and *Pparg* by binding to C/EBPβ [59].

Some probiotics exert cholesterol-lowering activity, favoring the prevention of cardiovascular diseases. *A. acidipropionici* C03B-STR isolated from goat milk degraded cholesterol in vitro, thus lowering its uptake by Caco-2 cells [86]. Cholesterol-lowering activity was demonstrated in vivo in mice and cows for dairy propionibacteria strains. These bacteria’s cholesterol-lowering mechanisms may include bile salt deconjugation, the co-precipitation of cholesterol with deconjugated bile salts, and excretion. Seven tested dairy propionibacteria strains were able to deconjugate sodium glycocholate, and two strains of *P. freudenreichii* and one *A. jensenii* strain also deconjugated sodium taurocholate. One *P. freudenreichii* strain, one *A. jensenii* strain, and one *A. thoenii* strain more efficiently supported cholesterol precipitation in the presence of oxgall [87].

*A. acidipropionici* OB7439 increased SCFA levels in the plasma, cecum, and feces of HFD-induced obese C57BL/6J mice. Moreover, administering this strain at high levels increased insulin secretion and consequently suppressed a rise in blood glucose after oral administration. These effects were strain-specific. Mice fed *A. acidipropionici* OB7439 showed a lower weight, a lower amount of white adipose tissue, and lower liver weight gain than the control after 16 weeks of administration. Plasma triglycerides and total cholesterol levels were also reduced. Based on previous research, the observed effects were attributed to increased propionate intake. The mRNA levels of *Tnfα*, macrophage marker *F4/80*, the fibrosis marker *Col*Iα, and *Fas* and *Chrebp* involved in fatty acid synthesis decreased in the liver. At the same time, those related to the peroxisome proliferator-activated receptor α *Ppara* involved in lipid metabolism increased. Energy regulation via the G protein-coupled receptor (GPR41) was involved in the observed effects, as demonstrated by the lack of similar outcomes in *GPR41*--/-- mice [60].

Table 3 summarizes the effects exerted in vivo by dairy propionibacteria in obese animal models.

### 3.4. Anti-Cancer Effects

*P. freudenreichii* DSM 20271 inhibited the proliferation of HCT116 CRC cells in a dose-dependent manner in vitro, as shown by the mitochondrial activity assay with 3--2,5-difeniltetrazolium bromide. In vivo, the effects exerted by this strain included the mitigation of oxidative stress induced by azoxymethane (AOM) in the colon of rats, as determined by measuring the concentration of malondialdehyde (MDA). Moreover, this strain reduced the formation of aberrant crypt foci induced by AOM to less than half, as well as the abnormalities observed in crypts at the cell and tissue levels. Supplementation with *P. freudenreichii* preserved the diversity of the intestinal microbiota compared to treatment with AOM alone. The enriched microbial groups differed from the control not treated with AOM and included lactobacilli and bifidobacteria [61].

The anticarcinogenic potential of an *A. acidipropionici* strain and a *P. freudenreichii* strain isolated from Swiss-type cheese could depend on their capacity to reduce intestinal β-glucuronidase activity, which releases toxic and carcinogenic compounds by hydrolyzing the glucuronic acid conjugates formed in the liver. This effect persisted in mice for one week after treatment with propionibacteria [62].

Moreover, *A. acidipropionici* CNRZ80, *P. freudenreichii* ITG18, and SI41 exerted an anticarcinogenic effect in vitro, which was attributable to the production of SCFAs that induced the apoptosis of cancer cells such as Caco-2, HeLa, and HT29. A medium fermented by *P. freudenreichii* CIRM BIA 138 induced death in HT29 colon cancer cells, and the optimal SCFA concentration was 0.75 g L^−1^ of acetate and 2.7 g L^−1^ of propionate [88]. Lan et al. showed that propionate induced the death of HT29 colon cancer cells via either apoptosis or necrosis in the pH range of 6.0–7.5 or at pH 5.5, respectively [89]. Moreover, *P. freudenreichii* TL133, able to survive and be metabolically active during transit in the gastrointestinal tract, also exerted this effect in the colons of rats exposed to the genotoxic agent 1,2-dimethylhydrazine (DMH). This chemical induced apoptosis in all crypt zones and different colon regions and an increase in the proliferative index. The administration of *P. freudenreichii* further increased the number of apoptotic cells, mostly in the middle crypt zone, but decreased the proliferative index [63].

TNF-related apoptosis-inducing ligand (TRAIL), a cytokine of the TNF superfamily, selectively kills cancer cells. To increase the effectiveness of TRAIL on CRC cells, synergistic treatments with chemotherapy agents or IFN-γ are required. As observed via DNA microarray analysis, culture supernatants or fermentation products from *P. freudenreichii* TL133 (CIRM BIA 138) induced 2180 genes in HT29 cells, and co-treatment with TRAIL increased the number of induced genes to more than three thousand. The genes induced by all the treatments included those encoding apoptosis pathways and immune response elements such as nucleotide-binding oligomerization domain (NOD)-like receptors and interactions between cytokines and receptors. These were more numerous for the association of TRAIL with fermentation products. Importantly, culture supernatants or fermentation products alone induced the TRAIL death receptor TRAIL-R2/DR5, which was found in the HT29 cell membrane, as shown via flow cytometry combined with antagonistic mouse monoclonal antibodies. The combination of TRAIL and *P. freudenreichii* culture supernatants or fermentation products induced 57% cell death in HT29 cells, but healthy epithelial colon cells HIEC were not sensitive to this treatment. *P. freudenreichii* culture supernatants or fermentation products, regardless of combination with TRAIL, induced the acetylation of histone H3, thus showing inhibitory activity for histone deacetylase. In addition, the expression of the apoptosis inhibitors FLIPL and XIAP decreased [63].

Milk and milk ultrafiltrate fermented by *P. freudenreichii* CIRM BIA 138 similarly induced the death of HT29 cells. The fermented milk ultrafiltrate induced caspases 3, -8, and -9. Apoptosis was confirmed via flow cytometry combined with propidium iodide staining, by the translocation of phosphatidylserine to the external layer of the cell membrane, the decrease in the mitochondrial membrane potential ΔΨm, the accumulation of superoxide anion O_2_^−^, and the release of cytochrome *c* into the cytoplasm. The changes induced in HT29 cells by TRAIL and *P. freudenreiichii* metabolites indicated that the extrinsic apoptotic pathway mediated by the activation of death receptors and caspase 8—as well as the intrinsic pathway, involving mitochondria and caspase 9—was activated [63].

Lectins are proteins or glycoproteins that bind to cell surface carbohydrates and are abundant in foods of plant origin. The lectins concanavalin A and peanut agglutinin stimulate the proliferation of epithelial cells in rat intestine and CRC cell lines and could have cancerogenic effects beyond an anti-nutritional function [90]. *A. acidipropionici* CRL 1198, a dairy strain able to survive in the gastrointestinal tract, when supplied together with concanavalin A, mitigated some of its detrimental effects in mice, such as epithelial cell proliferation in the intestine, structural alterations of microvilli, and an increase in enterobacteria and enterococci [64].

The lectins *Artocarpus integrifolia* agglutinin, soybean agglutinin, *Ulex europaeus* agglutinin, and concanavalin A induced the proliferation of cancer cell lines such as HCT-15, LoVo, SW837, and HT29 to different extents [90]. *A. acidipropionici* CRL 1198 was incubated in media containing the two most effective lectins, i.e., the *A. integrifolia* agglutinin and concanavalin A. The derived supernatants reduced the cell proliferation effect of lectins on SW837 cells, indicating the binding of these compounds by the propionibacteria. Adhesion to cells of *A. acidipropionici* was decreased in the presence of lectins and was restored after the addition of sugars able to bind to the lectins (haptens), indicating a lectin-binding capacity of *A. acidipropionici* mediated by cell surface carbohydrates [90].

Previous evidence that the SCFAs acetate, propionate, and butyrate favored the arrest of the cell cycle, the inhibition of histone deacetylase, and apoptosis in cancer gastric cells led us to test the effect of milk ultrafiltrate fermented by *P. freudenreichii* CIRM BIA 138 on HGT-1 cells. Fluorescence microscopy with Hoechst 33342 staining showed typical apoptotic nuclei and bodies in cells exposed to the fermented milk, with a higher degree of DNA fragmentation than the positive control, in which apoptosis was induced with campothecin. The fraction of cells in sub-G1 phase after treatment with the fermented milk was comparable to that observed with campothecin. Flow cytometry coupled with differential staining methods demonstrated the induction of apoptosis in up to 80% of the cells treated with fermented milk. In contrast, the number of necrotic cells was very low. The decrease in the ΔΨm membrane potential, the accumulation of the O_2_^−^ indicator of the production of reactive oxygen species (ROS), and cytochrome *c* release into the cytoplasm indicated apoptotic mitochondrial modifications. The activation of caspases 3, 8, and 9 occurred in HGT-1 cells exposed to the fermented milk and to a mixture of propionate/acetate at a ratio of 2:1. The *P. freudenreichii*-fermented milk doubled the cell-killing potential of campothecin [91].

*P. freudenreichii* DSM 20271 produced SCFAs in the culture medium used for CRC-derived RKO cells, most likely stimulated by the lactate produced by these cells via glucose fermentation (Warburg effect). Its culture broth inhibited the proliferation of CRC cells to different extents depending on the growth medium and previous adaptation to simulated digestive stress. Flow cytometry coupled with propidium iodide staining demonstrated the accumulation of CRC cells with cell-cycle arrest at the apoptotic sub-G1 phase or G2/M phase, indicating the inhibition of their proliferation [9].

To date, clinical studies on the indirect anti-cancer effects of dairy propionibacteria have only considered the association of *P. freudenreichii* JS/*L. rhamnosus* LC705 (Valio, Finland), which originated from cheese and is commonly used in semi-hard cheese production [92]. This strain association was shown to decrease the risk of hepatocellular carcinoma (HCC) in young Chinese men exposed to aflatoxin B1, which potentiates the liver cancer risk associated with hepatitis B virus (HBV) infection [93]. This strain association was previously shown to bind aflatoxin B1 and to decrease the level of this mycotoxin in the feces of healthy volunteers after two or three weeks of dietary supplementation [94]. In a double-blind trial on 90 subjects equally distributed in the intervention or the placebo group that did not significantly differ in the fecal concentration of the aflatoxin B1-N7-guanine (AFB-N7-guanine) marker of aflatoxin B1 exposure, probiotic administration for 5 weeks led to up to a 55% decrease in this marker in the urine, but this effect disappeared after the end of the intervention [93].

Another double-blind, placebo-controlled trial with the same bacterial association involved 38 men aged 24–55 years. It examined the effects on β-glucosidases, β-glucuronidases, and β-galactosidases, produced mainly by clostridia and *Bacteroides*, which release potentially carcinogenic compounds and urease, which is responsible for the formation of toxic and mutagenic substances from ammonia. The two bacterial strains did not cause adverse effects, and a 10% decrease in β-glucosidase activity in the intervention group was negatively correlated with the number of propionibacteria. A 13% decrease in urease activity, not correlating with the number of lactobacilli or propionibacteria, was also observed in the intervention group [92].

Table 4 summarizes the effects exerted in vivo by dairy propionibacteria on cancer prevention in animal models.

### 3.5. Effects of Propionibacterium freudenreichii on Bone Health

Rheumatoid arthritis is an autoimmune disease that causes chronic inflammation of the joints and can extend to most organs and the nervous system. This condition involves bone loss caused by enhanced osteoclast differentiation, and the available treatments do not support a definitive recovery [65,66]. The apoptosis regulator RANKL, a ligand of the RANK receptor activator of NF-kB, is one factor that stimulates osteoclast differentiation from macrophages [65,67]. Osteoclasts are multinucleated cells with a major role in bone destruction. They create an acidic microenvironment in which they secrete proteins involved in bone destruction, such as tartrate-resistant acid phosphatase (TRAP), cathepsin K (Ctsk), and calcitonin receptor (Calcr). Moreover, these cells produce pro-inflammatory cytokines and chemokines [65]. Osteoprotegerin (OPG) is a receptor that binds to RANKL and inhibits osteoclastogenesis by hindering the RANK-RANKL interaction. Heat-killed *P. freudenreichii* MJ2 inhibited osteoclastogenesis and mitigated collagen-induced arthritis (CIA) in a mouse model by increasing the *OPG*/*RANKL* expression ratio [67].

This effect was mediated by the surface proteins of *P. freudenreichii* MJ2 extracted with guanidine hydrochloride. Murine macrophages RAW 264.7 that can differentiate into osteoclasts were exposed to these proteins at a 10 μg/mL concentration for different days, and osteoclast differentiation was significantly inhibited. Moreover, TRAP+ osteoclasts decreased after 4 days of treatment with *P. freudenreichii* MJ2 surface proteins in a dose-dependent manner, as well as the expression of osteoclastogenic genes induced by RANKL and encoding RANK, c-fos, NFATc1, and NF-κB. Consequently, the genes regulated by the NFATc1 master regulator of osteoclast differentiation were expressed at lower levels [65].

The transcriptome of RAW 264.7 cells treated with *P. freudenreichii* MJ2 surface proteins comprised 888 differentially expressed genes, among which 128 genes were involved in osteoclast differentiation. Moreover, *lcn*2 encoding lipocalin 2, a protein with immunity function, showed the highest expression level, with 2426-fold upregulation. STRING protein–protein interaction and functional enrichment analysis highlighted that Lcn2 directly interacts with the tumor necrosis factor (*Tnf*), which, in turn, interacts with the *RANK* gene and the *Nfatc*1 gene, thus influencing the expression of downstream genes *Cst*1, *Cts*k, *Mit*f, and *TRAP*. Therefore, it was hypothesized that *lcn*2 might be involved in the inhibitory mechanism of *P. freudenreichii* MJ2 surface proteins on osteoclast differentiation [65].

The inhibition of *lcn*2 expression via small interfering RNA (siRNA) silencing led to TRAP activity, an increase in TRAP(+), and the formation of the F-actin ring responsible for bone resorption in cells treated with *P. freudenreichii* MJ2 surface proteins. This suggests that these proteins inhibit osteoclast differentiation by upregulating *lcn*2. Protein expression analysis showed that this effect was due to the downregulation of c-fos and NFATc1, along with their downstream genes. TRAP activity in osteoclasts was also decreased by the surface proteins of *P. freudenreichii* MJ2 treated at 100 °C for 30 min or treated with trypsin. In the latter case, TRAP activity inhibition was slightly enhanced, showing that the effect does not necessitate entire proteins. Liquid chromatography–tandem mass spectrometry (LC-MS/MS) separation of trypsin-treated surface proteins highlighted that chaperonins and heat shock proteins were their main components [65].

Whether the EVs from *P. freudenreichii* MJ2 were involved in the inhibition of osteoclastogenesis was investigated in a murine model of rheumatoid arthritis. The EVs produced by this strain had an average diameter of 171 nm. They contained 585 proteins involved in the metabolism, cell structure, and binding of various molecules, such as ATP, nucleotides, ions, carbohydrates, and cyclic and heterocyclic compounds. The EVs were not toxic to the RAW 264.7 cells even when produced by up to 8 Log CFU/mL of *P. freudenreichii*, so this level was used in the in vitro and in vivo experiments. *P. freudenreichii* EVs decreased RANKL-exposed RAW 264.7 murine macrophages’ differentiation into TRAP osteoclasts and resulted in significantly lower TRAP activity. Microscopy coupled with fluorescent antibodies showed reduced binding of RANKL to RANK in EV-treated cells [66].

*P. freudenreichii* EVs significantly decreased the arthritis score in mice with CIA that showed reduced bone erosion and synovial inflammation, a lower number of TRAP(+) osteoclasts, and collagen-specific IgGs. In addition, the levels of pro-inflammatory cytokines IL-6, TNF-α, and IL-17 decreased, while IL-10 increased in serum. The in vivo observations confirmed the decreased expression of genes associated with osteoclastogenesis and indicated an increase in the *OPG*/*RANKL* ratio. The *P. freudenreichii* EVs were not hepatotoxic, as indicated by unaltered aspartate transaminase (AST) and alanine transaminase (ALT) activities [66].

Table 5 reports the beneficial effects exein vivo by dairy propionibacteria on bone health with strains involved, animal models and active molecules.

## 4. Antimicrobial Properties of Dairy Propionibacteria

*P. freudenreichii* JS, a strain with a high capacity to adhere to intestinal mucus, prevented the adhesion of *Staphylococcus aureus* RN4220 by 39% and significantly reduced the viability of adhered *S. aureus* to 72% by producing non-bacteriocin antimicrobial substances [95]. A pathogen inhibitor produced by *P. freudenreichii* PTCC 1674 is a surfactant/emulsifier lipopeptide showing slight inhibition and adhesion prevention in *E. coli*, *Pseudomonas aeruginosa*, *S. aureus*, and *B. cereus* in culture plates [96]. Dairy propionibacteria were reported to inhibit *E. coli* and *Shigella sonnei* strains in vitro, but not *L. monocytogenes*, as an effect of the low pH of the growth medium [73]. Two *P. freudenreichii* dairy strains, B3523 and B4327, showed high percentages of adhesion to budgerigar abdominal tumor cells (BATCs), and their cell-free culture supernatants inhibited multidrug-resistant (MDR) *S. heidelberg*, *E. coli* O157:H7, and *L. monocytogenes*. The *P. freudenreichii* strains did not invade BATCs and were not hemolytic [97].

The strain *P. freudenreichii* B3523 exhibited inhibitory activity toward three *Salmonella* serovars, *S. enteriditis, S. typhimurium, and S. heidelberg, by reducing their motility, adhesion, and the invasion of avian epithelial cells, as well as their multiplication in the cecal conte*nts of turkeys. The *P. freudenreichii* strain adhered to epithelial cells, and its cell-free extracts inhibited the growth and motility of *S.* Heidelberg [98]. When this strain was supplied at about 10 Log CFU daily to turkey poults of 2 to 12 weeks old challenged with the MDR *S. heidelberg* GT2011, a foodborne strain that caused an outbreak from turkey meat [69], the pathogen levels in the cecal content were reduced by 1 to 2 Log CFU/g. This effect was linked to the changes induced by *P. freudenreichii* in the intestinal microbiota composition of growing poults, namely, the increase in actinobacteria and the genus *Subdoligranulum* and the decrease in *Streptococcus*. This effect was observed after 2 days of treatment but not later. In finishing turkeys, supplementation with *P. freudenreichii* increased lactobacilli and Ruminococcaceae after 2 days and led to an increase in the genera *Lactococcus*, *Erysipelatoclostridium*, *Leuconostoc*, and *Butyricicoccus* after 7 days. In contrast, the *S. heidelberg* control group showed a higher abundance of *Turicibacter* and *Streptococcus*, varying in different groups and with different sampling times. Similar results were obtained for finishing turkeys in a separate experiment. The genera enriched in the *P. freudenreichii* treatment groups are responsible for the formation of SCFAs that promote gut health. In contrast, the genus *Streptococcus* comprises opportunistic pathogens, and the genus *Turicibacter* is associated with intestinal inflammatory diseases. Hence, the results indicate a protective effect of *P. freudenreichii* against intestinal dysbiosis induced by the *S. heidelberg* pathogen [68].

It was later reported that in treatments of turkeys with *P. freudenreichii* B3523, the dissemination of *S. heidelberg* in the liver and spleen was reduced from 20% to 60% depending on the age of animals and treatment time [69]. The same *P. freudenreichii* strain was used to treat turkeys challenged with drug-resistant field turkey isolates of *S.* Agona, *S.* Saintpaul, and *S.* Reading. It was proven to be as effective as a *Salmonella* vaccine in reducing the pathogen in the cecum and was even more effective when combined with the vaccine. All the liver and spleen samples from animals receiving the vaccine and *P. freudenreichii* were negative for *Salmonella*, even though *P. freudenreichii* alone showed a relatively low percentage of *Salmonella-negative* organ samples. The reason for the higher efficacy of the *P. freudenreichii*/vaccine combination compared with the vaccine alone and the vaccine combined with a *Ligilactobacillus salivarius* strain was not investigated [70].

*A. acidipropionici* Q4, isolated from an Argentinian Swiss-type cheese, adhered to HT29 cells and prevented the adhesion of *E. coli* and *S*. Enteriditis, mediated by its cell surface proteins (CSP). These separately exerted similar effects [99].

*A. jensenii* B-6085 and *A. thoenii* B-6082 showed moderate inhibitory activity against the intestinal pathogens *E. coli* ATCC 25922, *S. enterica* ATCC 14028, *S. aureus* ATCC 25923, *P. aeruginosa* B6643, *Proteus vulgaris* ATCC 63, and *L. monocytogenes* ATCC 7644, and the strain *P. freudenreichii* B-11921 showed weaker activity compared to the *Acidipropionibacterium* spp. against *L. monocytogenes* [100].

*P. freudenreichii* DSM 20271 and SS10 (Optim PropioniBacter, Laboratoire Optim, Bionoto sprl., Belgium), as well as *A. acidipropionici* DSM 20272, showed very slight inhibiting effects against wound pathogens in vitro, confirming previous results found for these species. However, moderate antimicrobial activities were previously reported for *A. jensenii* B-6085 and *A. thoenii* B-6082 [101].

The cheese isolates *P. freudenreichii* AS2 and AS51, as well as the malt isolate *A. virtanenii* JS278, exerted anti-QS activity with the consequent inhibition of biofilm formation for *Chromobacterium violaceum* ATCC 31532, with *P. freudenreichii* exerting the most pronounced effect. The comparison with a deletion mutant for the gene *cvil* for QS-dependent biofilm formation highlighted that this activity depended on the inhibition of the synthesis of the auto-inducer (AI) acyl-homoserine lactone (AHL) by acetic and propionic acid [102].

## 5. Production of Beneficial Metabolites

The role of *A. jensenii* 702 in correcting B12 vitamin deficiency in vivo was demonstrated in rats fed a diet deficient in B12 for three months. Supplementation with 10 Log CFU of the bacterium daily for two months restored the B12 vitamin levels found before administration of the B12 vitamin-deficient diet. After three months of *A. jensenii* 702 administration, the B12 vitamin levels were comparable to those obtained by directly supplementing the vitamin, showing that its deficiency can be corrected by supplying the bacterium through fermented food [103]. A contrasting result was obtained with the generally recognized as safe (GRAS)-certified strain *P. freudenreichi* ATCC 6207, a vitamin B12 producer. This was added in 8 Log CFU/g to yogurt administered to 30 volunteers, 18 to 50 years old, at the KEM Hospital, Pune, India, in a double-blind placebo trial aimed at preventing vitamin B12 deficiency. No significant differences were found in the vitamin B12 concentration in venous blood samples in the *P. freudenreichii* group compared to the placebo group. The causes for the missing beneficial effect were not investigated [104].

Four *P. freudenreichii* isolates from goat milk, selected based on growth capacity and tolerance to gastrointestinal and technological stresses, were used to ferment the Scotta dairy product from two industries. The formation of vitamin B9 vitamers, cobalamin derivatives, and folates was demonstrated, and the amounts differed between the two dairies [15]. A *P. freudenreichii* PS-4 strain (Christian Hansen, Denmark) was used for the in situ production of conjugated linoleic acid (CLA), a substance with antioxidant, anti-cholesterolemic, anti-inflammatory, and anti-carcinogenic properties, at levels up to 6.4 mg/g of fat in a yogurt supplemented with inulin [105]. Studies on fermented food products of both plant and animal origin, such as sourdough bread, tofu, and fermented milk, have demonstrated the potential of dairy propionibacteria, singly or in combination, to enrich the diet with vitamin B12, SCFAs, folate, and bioactive peptides [106,107,108,109,110,111,112]. Moreover, *A. acidipropionioci* LET 120, a strain with high β-galactosidase activity, produces prebiotics from lactose and lactulose, namely, oligosaccharides from lactose (GOS) and lactulose (OsLu). The *P. freudenreichii* strain DSM 4902 was utilized to produce vitamin B12 on spent beer yeast [113,114].

## 6. Safety of Dairy Propionibacteria

The only known virulence factor of dairy propionibacteria is β-hemolytic activity, which is expressed by red-pigmented strains of the species *A. jensenii* and *A. thoenii* that produce granadaene. Adverse events caused by granadaene-producing strains have never been reported, but these should preferably be absent in food. No detrimental activities, such as biogenic amine formation in cheese, have been reported for dairy propionibacteria [115,116]. Granadaene production in *A. jensenii*, *A. thoenii*, and *A. vitanenii* is encoded by the *cyl* gene cluster comprising the genes *acpC*, *cylZ*, *cylA*, *cylB*, and *cylE*, homologous to those found in *S. agalactiae* and essential for hemolytic activity. The link between the red pigmentation and hemolytic activity was confirmed for *A. jensenii*, *A. thoenii*, and *A. virtanenii* strains, while non-pigmented strains, including *P. freudenreichii* strains, did not show any hemolytic activity. A conserved region in the *cyl*G gene was selected for PCR-based diagnosis of hemolytic activity in dairy propionibacteria [116].

No safety issues were reported for the strains tested as probiotics. In particular, *A. jensenii* 702 was fed to rats for 81 days without causing adverse effects, and it did not affect body and organ weights and fecal β-glucuronidase activity. Moreover, extra-intestinal translocation was not observed [70]. This strain was tested as DFM in calves to increase propionate and butyrate in the rumen and support an increase in feed conversion and growth rate. The group of calves treated with *A. jensenii* 702 showed a faster and statistically significant weight gain until 18 weeks after treatment. No adverse effects of the supplied propionibacteria emerged from hematological values [74]. The same strain supplied to layer chickens in doses of 7 Log CFU per day led to an increase of 4.2% in egg weight with no adverse effects [75]. *A. acidipropionici* LET105 and LET107 isolated from chicken intestine were administered daily to 1–14-day-old chicks in a 6 Log CFU dose with no adverse effects or difference in growth rate. The group of animals receiving the propionibacteria showed better development of the intestinal mucosa with increased crypt length, goblet cell number, and mucus production [13]. *A. acidipropionici* P169 used as DFM in Holstein calves post-partum led to a dose-dependent increase in milk yield and weight loss reduction, along with a general improvement in health status [73].

The culture medium of *P. freudenreichii* ET-3, produced in the form of tablets by Meiji and able to selectively stimulate the growth of bifidobacteria in the human gut, when supplied to rats in high amounts, i.e., 6 g per kg per day, did not cause any clinical sign of toxicity for different organs at the macroscopic and microscopic levels, and no alterations were observed in hematological and chemical values. Moreover, the preparation was not mutagenic for *Salmonella* spp. and *E. coli* indicators and Chinese hamster lung cells [117]. Clinical safety assessments for participants receiving the culture medium for one week showed no statistically different hematological and chemical parameters compared to the placebo group and the baseline. In participants receiving the culture medium for 13 weeks, there was a significant decrease in total blood protein levels, white blood cells, hemoglobin, and mean corpuscular hemoglobin concentration, as well as an increase in red cells’ medium volume and urine pH, but the values remained in the normal ranges. No gastrointestinal symptoms could be attributed to the *P. freudenreichii* culture medium. Therefore, no adverse effects were attributed to its dietary supplementation [118]. *P. freudenreichii* JS, in combination with *L. rhamnosus* GG, did not induce gastrointestinal or respiratory disorders or infections in randomized, double-blind, placebo-controlled clinical studies conducted on 1909 healthy participants [119].

Regarding antibiotic resistance, it was reported that *P. freudenreichii* strains showed intrinsic resistance to aminoglycosides, quinolones, including levofloxacin, oxacillin, metronidazole, and kanamycin, and no transferable antibiotic resistance was described based on plasmid-curing experiments [115]. Phenotypic resistance tests carried out via disk diffusion for the antibiotics ampicillin, benzylpenicillin, carbenicillin, polymyxin, streptomycin, gentamicin, clotrimazole, chloramphenicol, tetracycline, neomycin, and kanamycin indicated *P. freudenreichii* B-11921’s moderate resistance to gentamicin and neomycin, sensitivity to polymixin, and resistance to all the other antibiotics. The tests indicated *A. acidipropionici* B-5723’s moderate resistance to benzylpenicillin, gentamicin, and clotrimazole; sensitivity to polymyxin and tetracycline; and resistance to all the other antibiotics. The tests indicated *A. jensenii* B-6085’s moderate resistance to carbenicillin, polymyxin, and tetracycline and high resistance to all the other antibiotics. Finally, the tests also indicated *A. thoenii* B-6082’s moderate resistance to streptomycin, clotrimazole, and chloramphenicol and resistance to all the other antibiotics [100]. However, the genetic bases of resistance were not investigated, and no reference cut-off values were known or determined for these bacteria.

Testing using the broth microdilution method according to the ISO 10932:2010 norm [120] indicated that 47 dairy propionibacteria isolated from goat milk, cheese, and rumen in Sardinia Island, Italy, were resistant to amoxicillin. For the species *P. freudenreichii*, 71%, 48%, 24%, and 13% of the isolates were resistant to spectinomycin, ciprofloxacin, erythromycin, and tetracycline, respectively, while 4 isolates were resistant to kanamycin. Among 18 *Acidipropionibacterium* strains, 7 were resistant to tetracycline, 3 to ciprofloxacin, 2 to kanamycin, and 1 to clindamycin, according to the cut-off values fixed for bifidobacteria and non-enterococcal lactic acid bacteria [121]. Four *P. freudenreichii* strains and one *A. acidipropionici* strain showed multiple antibiotic resistance, and only three strains of *P. freudenreichii* and six *A. acidipropionici* strains did not show any antibiotic resistance [15]. The phenotypic resistance of some *Propionibacterium* and *Acidipropionibacterium* strains was reported toward vancomycin and ciprofloxacin, and reversible resistance was observed for tetracycline [73]. The lectin-binding strain *A. acidipropionici* LET103, intended for use as an avian growth promoter in a multi-strain preparation, did not express virulence factors or antibiotic resistance [122].

The only known genetic traits involved in antibiotic resistance in dairy propionibacteria are the non-transferable mutations G2294A and G2295A in the 23S rRNA that possibly determine resistance to macrolide antibiotics observed in *P. freudenreichii* T82 [123] and genes encoding a mitomycin radical oxidase; a protein with a tetracycline repressor domain; and a puromycin resistance protein, Pur8, found on a genomic island in *P. freudenreichii* JS8 [2].

## 7. Discussion

Based on the studies consulted for this review, dairy propionibacteria can have a wide spectrum of applications as probiotics, with proven benefits in IBDs, obesity, rheumatoid arthritis, allergies, and infections in animal models [14,43,44,45,46,47,48,49,50,51,52,53,54,55,56,57,58,59,60,61,62,63,64,65,66,67,68,69,70]. Despite these bacteria being less commonly investigated than lactobacilli and bifidobacteria, they also demonstrated multiple disease-mitigation activities often associated with the activation of anti-inflammatory components of the immune system. The capacity to produce vitamin B12, folate, and CLA represents an additional benefit that could be derived from supplementing substrates or foods fermented by dairy propionibacteria. However, in the case of vitamin B12, studies aimed at evaluating the effect of living dairy propionibacteria in supplying this vitamin had contrasting findings that could be explained by the inability of some strains to synthesize it without an external supply of the 5,6-dimethylbenzimidazole (DMBI) ligand [3]. Therefore, efficient B12 vitamin producers should be selected according to standardized methodological approaches while considering this aspect.

**Figure 1:**
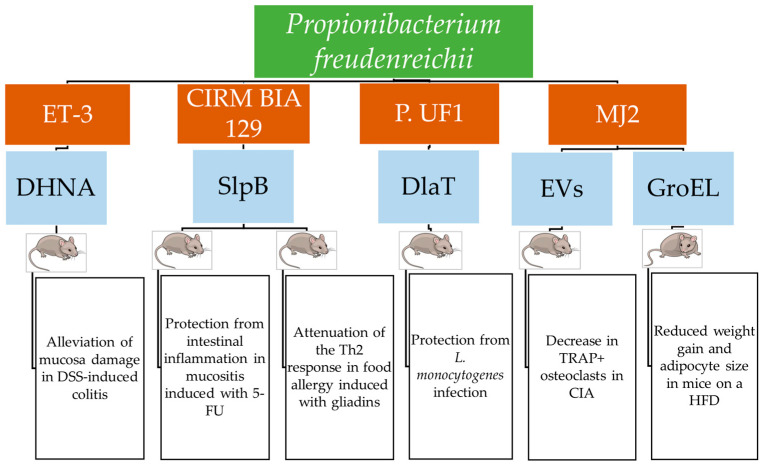
Molecules involved in the probiotic action of *P. freudenreichii*, with the effects proven in vivo [14,44,45,52,56,58,59,66]. DSS: sodium dextran sulfate; 5-FU: 5-fluorouracile; CIA: collagen-induced colitis; HFD: high-fat diet.

Some of the studies led to the in vivo identification of the molecules produced by dairy propionibacteria and that are responsible for the probiotic effects. The specific strains, molecules, and effects are schematized in Figure 1.

**Figure 2:**
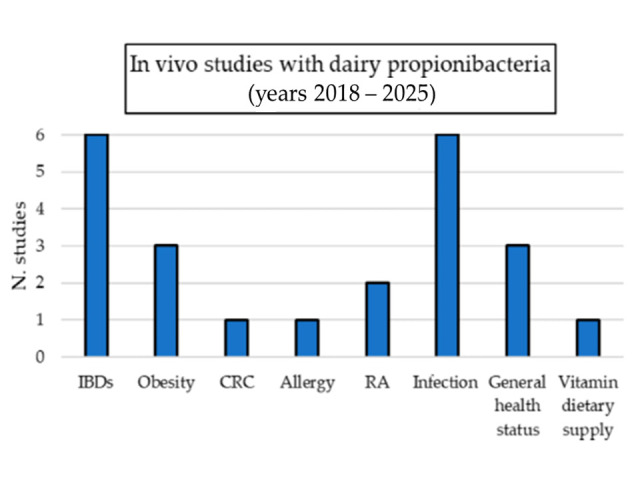
The number of in vivo studies evaluating the probiotic properties of dairy propionibacteria published since 2018 [49,50,51,52,53,54,55,56,59,60,61,66,67,68,69,70,80,83,100,104] after the latest review article by Rabah et al. [24]. IBDs: inflammatory bowel diseases; CRC: colorectal cancer; RA: rheumatoid arthritis.

Figure 2 shows the number of in vivo studies carried out since 2018, when the latest review on the probiotic properties of dairy propionibacteria was published.

Only 23 new in vivo studies involved these bacteria, an average of less than 3 per year, thus showing that they are largely understudied and underexploited despite the promising results obtained in most investigations. The opportunity to conduct new studies on the dietary supplementation of dairy propionibacteria should be encouraged by the total absence of infection case reports with their involvement, unlike that found for lactobacilli [124], bifidobacteria [125], and the widely used probiotic *Saccharomyces boulardii* [126,127]. This might depend on their less frequent use in living probiotic supplementation for humans, which was limited to the strain *P. freudenreichii* JS [92]. However, it must be noted that dairy propionibacteria are frequently present in cheeses, naturally or intentionally added, and adverse effects have never been reported for this exposure source. Other evidence supporting the incapability of dairy propionibacteria to be harmful is the absence of adverse effects and extraintestinal translocation in farm animals and animal disease models [70]. Moreover, studies evaluating the toxicity of dairy propionibacteria did not report any adverse effects, and those studying survival in the gastrointestinal tract never indicated long persistence after the administration period, which could be a consequence of transient adhesion to intestinal epithelial cells or mucus. The inability of *P. freudenreichii* to invade intestinal epithelial cells was demonstrated in vitro [37,97]. All these indications should encourage further clinical trials to explore the possible beneficial effects of dairy propionibacteria for humans. Possible ambits for clinical evaluation are randomized controlled trials on participants affected by the conditions already successfully treated in animal models with strains of dairy propionibacteria with no adverse effects.

The use of active molecules rather than living propionibacteria could also be considered. Indeed, studies on *P. freudenreichii* ET-3 supplied as a culture medium concentrate and not as a living probiotic demonstrated that producing postbiotics from dairy propionibacteria is also possible. Further indications supporting this possibility are the beneficial effects obtained using the purified immunomodulatory proteins or EVs comparable with living bacteria in animal hosts [14,39,43,46,47,48,49,52,56,58,59,66].

The molecular basis of dairy propionibacteria interactions with the immune system of host cells and tissues was clarified for strains of the species *P. freudenreichii* able to induce host responses, which led to strain-dependent inflammation mitigation and tissue integrity protection. In particular, the gene encoding SlpB, one of the proteins with the highest immunomodulatory effect produced by *P. freudenreichii*, was found in the genome of only 2 among 20 *P. freudenreichii* strains included in a genome sequencing and comparison study [2]. Other proteins involved in the probiotic effects of *P. freudenreichii* were the central metabolism protein DlaT and the chaperonin GroEL [14,56]. These proteins belong to the core genome of bacteria and, in the case of *P. freudenreichii*, they only function as mediators of probiotic activities in some strains. This could be explained by their overexpression, reflecting the presence of more than one gene copy, or rearrangements in the regulatory regions that lead to increased expression. The genome duplication observed in some *P. freudenreichii* strains could be at the origin of the increased expression of some intracellular proteins more abundant in cell surface protein extracts or EVs [2].

Identifying the protein mediators of probiotic activity enables the design of screening tests targeting their encoding genes for selecting new *P. freudenreichii* strains that can be used to promote health benefits, based on expression levels determined using reverse transcription quantitative PCR tests (RT-qPCR). Similarly, tests aimed at determining the *gtf* gene expression level could enable the exclusion of strains whose surface proteins are likely to be shielded by a polysaccharide capsule [30].

In one *P. freudenreichii* strain, among twenty examined, a pilus structure was found to be expressed and promote binding to mucus [2]. Since this character is known as a main factor favoring adhesion and, if overexpressed, can even increase the capability of probiotics to cause infections [124], its occurrence in dairy propionibacteria and its involvement in persistence in the gastrointestinal tract should be evaluated.

No molecular investigations were conducted for the *Acidipropionibacterium* genus despite the health-promoting effects observed in animal trials and farm animals and the promising results obtained for *A. acidipropionici* OB7439 in an HFD mouse model [60]. There is currently a strong disproportion between the number of studies regarding *P. freudenreichii* compared to the other dairy propionibacteria and a knowledge gap regarding the probiotic potential of *Acidipropionibacterium* spp. that should be better investigated by acquiring a large number of genome sequences to identify functional traits, ensure safety, and possibly expand their application as food and feed supplements.

From a “one health” perspective, studies on attenuating *Salmonella enterica* infections in turkeys demonstrated that dairy propionibacteria can reduce the risk of human exposure to zoonotic pathogens by decreasing the infection levels in farm animals [68,69]. Therefore, this research field should be continued.

To support the safe use of dairy propionibacteria as probiotics, the study of the genetic bases for the antibiotic resistance phenotypes observed in some studies and the definition of ecological cut-off (ECOFF) values of antibiotic resistance by examining a large number of isolates should be completed. This could support a more reliable distinction between intrinsic and acquired resistance than is currently possible. Indeed, though no transferable antibiotic resistance was reported for these species, up-to-date studies focused on this aspect are suggested due to the risk of antibiotic resistance gene transfer from probiotic to pathogenic bacteria [128].

## 8. Conclusions

Dairy propionibacteria are a component of the naturally occurring microbiota in cheeses produced with raw milk or added as selected cultures in industrial Swiss-type cheeses. Infections caused by these bacteria in humans or animals have never been reported, and their probiotic activities, demonstrated at the molecular level and in vivo, may help in a variety of different disease conditions. Based on current knowledge, dairy propionibacteria exert beneficial effects on IBDs, obesity, rheumatoid arthritis, immunomodulation, CRC, and infection mitigation in vivo, but these effects require evaluation in clinical studies. In addition, dairy propionibacteria can enrich foods or culture media with their main metabolites; the SCFAs propionate and acetate, which have been shown to favor apoptosis in cancer cell lines; vitamin B12; folate; and CLA. However, they are not yet widely exploited as commercial probiotics or in probiotic foods. The molecular basis of the probiotic properties of *Acidipropionibacterium* spp. and the development of adequate procedures to evaluate phenotypic and genotypic antibiotic resistance in these bacteria require further investigation.

## Figures and Tables

**Table 1:** Dairy propionibacteria’s beneficial in vivo effects on intestinal inflammation: the bacterial strains, disease models, animal hosts, and molecules involved.

Strain	Disease	Animal Model	Active Molecule	Induced Effects	Reference
*P. freudenreichii* ET-3	TNBS-induced colitis	Rats	Propionate	Ulcer healing	[43]
*P. freudenreichii* ET-3	DSS-induced colitis	Mice	DHNA	Reduction in molecules favoring leucocyte infiltration and vascular adhesion. Downregulation of IL-1β, IL-6, and TNF-α. Increase in lactobacilli and SCFAs. Activation of AhR-regulated pathways. Increase in colitis suppressing C-type RegIII lectins	[44,45]
*P. freudenreichii* CIRM BIA129	TNBS-induced colitis	Mice	Cell surface proteins	DAI reduction. Attenuation of inflammation (increased expression of *Pparγ*) and oxidative stress (decreased expression of *cox*2 and *Hmox*). Restoration of intestinal barrier (increased expression of ZO-1). Decrease in IL-1β, IL-6, and IL-17	[39,46,47,48,49]
*P.freudenreichii* KCTC 1063	DSS-induced colitis	Rats	SCFAs	DAI reduction. Reduced crypt damage and leucocyte infiltration. Maintenance of mucin MUC2 expression level	[50]
*P. freudenreichii * B1	DSS-induced colitis	Mice	Not investigated	Decrease in IL-8, IL-1β, and TNFα. Increase in ZO-1, claudin-1, and Rspo3. Downregulation of RHO kinase ROCK-1, and Axin2, which inactivate Wnt/β-catenin regeneration pathway.	[51]
*P. freudenreichii* CIRM BIA 129	Mucositis induced with 5-FU	Mice	SlpB	Reduction in leucocyte infiltration and ulceration. Restored height of villi. Reduction in gut permeability (increased expression of *cld*1). Decreased expression of IL-17a, IL-12, and IL-1β	[52]
*P. freudenreichii* P.UF1	NEC-like injury	Newborn mice	DlaT	Downregulation of nitric oxide synthase *iNOS* and interleukins *Il-1b*, *Il-6*, and *Il-23.* Increase in ILC3 expressing IL-17A and IL-22	[14]
*P. freudenreichii* P.UF1	Infection with *L. monocytogenes*	Mice	DlaT	Reduction in IL-1β, IL-6, and IL-12/IL23p40 produced by DCs. Reduction in Th1 cells producing IFNγ and increase in Th17 cells and IL-10^+^ Treg cells.	[14]

TNBS, 2,4,6-trinitrobenzenesulfonic acid; DSS, sodium dextran sulfate; DHNA, 1,4-dihydroxy-2-naphtoic acid; SCFAs, short-chain fatty acids; DAI, disease activity index; 5-FU, 5-fluorouracile; NEC, necrotizing enterocolitis; DCs, dendritic cells.

**Table 2:** The in vivo immunomodulation effects of dairy propionibacteria: the bacterial strains, disease models, animal hosts, and molecules involved.

Strain	Disease	Animal Model	Active Molecule	Induced Effects	Reference
*A. jensenii* 702	Administration of *M. tuberculosis* culture filtrate	Rats	Not defined	IFNγ levels 2 to 3 Log higher than IL-4, indicative of Th1 response effective in protection from tuberculosis	[54]
*P. freudenreichii* KCTC 1063	No disease	*C. elegans*	Not defined	Upregulation of innate immunity-related pathways DBL/TGF-β, P38 MAPK, and Daf-2/DAF-16 involved in IIS; upregulation of antimicrobial peptide genes *lys-7* and *lys-8*	[55]
*P. freudenreichii* CIRM BIA 129	Food allergy induced with wheat gliadins	Mice	SlpB	Prevention of body temperature increase; prevention of gliadin-specific IgE and IgG1 increase in serum; increase in gliadin-specific IgG2; prevention of intestinal permeabilization	[56]

**Table 3:** In vivo effects of dairy propionibacteria on mitigating obesity: bacterial strains, animal hosts, and molecules involved.

Strain	Disease	Animal Model	Active Molecule	Induced effects	Reference
*P. freudenreichii* JS	HFD-induced obesity	ApoE*3 Leiden transgenic mice	Not investigated	Reduced weight gain and gonadal adipose tissue Decreased levels of VCAM-1 vascular inflammation marker Decreased mast cell number and TNF-α levels	[57]
*P. freudenreichii* MJ2	HFD-induced obesity	Mice	GroEL	Reduced fat accumulation Pre-adipocyte stage maintenance by upregulation of Pref-1 and downregulation of PPARγ, C/EBPα, FAS, SCD-1, ACC, and lipolytic enzymes	[58,59]
*A. acidipropionici* OB7439	HFD-induced obesity	Mice	Not investigated	Increased insulin secretion Decreased *Tnfα*, *F4/80*, *Col*Iα, *Fas*, and *Chrebp*; increased *Ppara* in liver	[60]

HFD: high-fat diet.

**Table 4:** The in vivo cancer prevention effects of dairy propionibacteria: bacterial strains, animal hosts, and observed responses.

Strain	Carcinogen	Animal Model	Induced Effects	Reference
*P. freudenreichii* DSM 20271	AOM	Rats	Reduced formation of aberrant crypt foci	[61]
*P. freudenreichii* TL133	DMH	Rats	Increased apoptosis and decreased proliferation of crypt cells	[89]
*A. acidipropionici* CRL 1198	Concanavalin A	Mice	Reduced proliferation of intestinal epithelial cells; preserved microvilli structure	[90]

AOM: azoxymethane; DMH: dimethylhydrazine.

**Table 5:** The beneficial in vivo effects of dairy propionibacteria in bone diseases, as well as the bacterial strains involved, animal models, and active molecules.

Strain	Disease	Animal Model	Active Molecule	Induced Effects	Reference
*P. freudenreichii* MJ2	CIA	Mice	Surface protein extracts	Increased *OPG*/*RANKL* expression ratio	[64,65]
*P. freudenreichii* MJ2	CIA	Mice	EVs	Decrease in IL-6, TNF-α and IL-17 Increase in IL-10 Increased *OPG*/*RANKL* expression ratio	[66]

CIA: collagen-induced arthritis; EVs: extracellular vesicles.

## Data Availability

No new data were created.

## Abbreviations

The following abbreviations are used in this manuscript:

ACNQ	2-amino-3-carboxy-1,4-naphthoquinone
ALT	Alanine aminotransferase
BALs	Bovine alveolar lavage cells
BCFA	Branched-chain fatty acid
CFU	Colony-forming unit
CRISPR-Cas	Clustered regularly interspaced short palindromic repeats
CW	Cell wall
DAI	Disease activity index
DBL	DNA-binding ligand
DC	Dendritic cell
DFM	Direct fed microbial
DHNA	1,4-dihydroxy-2-naphtoic acid
DSS	Dextran sulfate
DTPA	Diethylenetriamine pentaacetic acid
EFSA	European Food Safety Authority
ELISA	Enzyme-linked immunosorbent assay
EPS	Exopolysaccharide
EVs	Extracellular vesicles
FIL-IDF	International Dairy Federation
FITC	Fluorescein isothiocyanate
5-FU	5-fluorouracile
GIT	Gastrointestinal tract
GRAS	Generally recognized as safe
HDL	High-density lipoprotein cholesterol
HFD	High-fat diet
HGT	Horizontal gene transfer
HOMA-IR	Homeostasis model assessment insulin resistance
HRP	Horseradish peroxidase
IBD	Inflammatory bowel disease
ICE	Integrative–conjugative element
IEB	Intestinal epithelial barrier
IFN-γ	Interferon γ
Ig	Immunoglobulin
IHC	Immunohistochemistry
IL	Interleukin
LDL	Low-density lipoprotein cholesterol
LPS	Lipopolysaccharide
MAdCAM-1	Mucosal addressin cell adhesion molecule 1
MAPK	Mitogen-activated protein kinase
MHC II	Major histocompatibility complex II
MLNC	Mesenteric lymph node immune cell
mMCP-1	Mucosal mast cell protease-1
MoDC	Monocyte-derived DC
MSCs	Mesenchymal stem cells
MTT	3-[4,5-dimethylthiazol-2-yl]-2,5-diphenyltetrazolium bromide
NEC	Necrotizing enterocolitis
NF-kB	Nuclear factor kappa-light-chain-enhancer of activated B cells
NK	Natural killer
PBMCs	Peripheral blood mononuclear cells
QPS	Qualified presumption of safety
qRT-PCR	Quantitative reverse transcriptase polymerase chain reaction
SCFA	Short-chain fatty acid
SDS-PAGE	Sodium dodecyl sulfate polyacrylamide gel
SLH	Surface layer homology
Slp	S-layer protein
SP	Surface protein
Th	T helper
TEER	Trans-epithelial electrical resistance
TGF-β	Tumor growth factor-β
TNBS	2,4,6-trinitrobenzenesulfonic acid
TNF-α	Tumor necrosis factor α
Treg	Regulatory T cell
UC	Ulcerative colitis
UF	Ultra-filtered
XRE	Xenobiotic-responsive element
YEL	Yeast extract lactate
